# Parasite-Host Dynamics throughout Antimalarial Drug Development Stages Complicate the Translation of Parasite Clearance

**DOI:** 10.1128/AAC.01539-20

**Published:** 2021-03-18

**Authors:** Lydia Burgert, Sophie Zaloumis, Saber Dini, Louise Marquart, Pengxing Cao, Mohammed Cherkaoui, Nathalie Gobeau, James McCarthy, Julie A. Simpson, Jörg J. Möhrle, Melissa A. Penny

**Affiliations:** a Swiss Tropical and Public Health Institute, Basel, Switzerland; b University of Basel, Basel, Switzerland; c Centre for Epidemiology and Biostatistics, Melbourne School of Population and Global Health, University of Melbourne, Melbourne, Australia; d QIMR Berghofer Medical Research Institute, Brisbane, Australia; e School of Mathematics and Statistics, University of Melbourne, Melbourne, Australia; f Medicines for Malaria Venture, Geneva, Switzerland

**Keywords:** antimalarial drug development, translational medicine, parasite-host interactions, parasite clearance, within-host models

## Abstract

Ensuring continued success against malaria depends on a pipeline of new antimalarials. Antimalarial drug development utilizes preclinical murine and experimental human malaria infection studies to evaluate drug efficacy. A sequential approach is typically adapted, with results from each stage informing the design of the next stage of development. The validity of this approach depends on confidence that results from murine malarial studies predict the outcome of clinical trials in humans. Parasite clearance rates following treatment are key parameters of drug efficacy. To investigate the validity of forward predictions, we developed a suite of mathematical models to capture parasite growth and drug clearance along the drug development pathway and estimated parasite clearance rates. When comparing the three infection experiments, we identified different relationships of parasite clearance with dose and different maximum parasite clearance rates. In Plasmodium berghei-NMRI mouse infections, we estimated a maximum parasite clearance rate of 0.2 (1/h); in Plasmodium falciparum-SCID mouse infections, 0.05 (1/h); and in human volunteer infection studies with P. falciparum, we found a maximum parasite clearance rate of 0.12 (1/h) and 0.18 (1/h) after treatment with OZ439 and MMV048, respectively. Sensitivity analysis revealed that host-parasite driven processes account for up to 25% of variance in parasite clearance for medium-high doses of antimalarials. Although there are limitations in translating parasite clearance rates across these experiments, they provide insight into characterizing key parameters of drug action and dose response and assist in decision-making regarding dosage for further drug development.

## Abstract (precis)

Ensuring continued success against malaria depends on a pipeline of new antimalarials. Antimalarial drug development utilizes preclinical murine and experimental human malaria infection studies to evaluate drug efficacy.

## TEXT

Recent progress in reducing malaria burden is threatened by emerging resistance against current first-line treatments and by suboptimal adherence to existing treatment schedules. Development of new antimalarial treatments is therefore more urgent than ever (1). Requirements for new antimalarial treatment regimens are multifaceted, spanning safety, efficacy, and dose optimization for all populations, including pregnant women, infants, and children (2).

Before testing compounds *in vivo*, promising compounds are identified *in vitro*, and their parasiticidal efficacy is assessed in whole-cell or target-based assays (3). In preclinical stages, drug efficacy is often investigated using murine malarial infection. Historically, infection of normal mice with Plasmodium berghei was shown to be a useful experiment to measure crude drug efficacy and to select promising candidates (4). However, enzymatic differences between the human malaria parasite Plasmodium falciparum led to selection bias in candidate selection (5). More recently, the humanized mouse model of NOD*^scidIL-2R' c^*^−/−^ (SCID) mice infected with P. falciparum has been shown to provide insights into efficacy against the human parasite *in vivo* (6–8) and further assists candidate selection. Early human efficacy studies via experimental infection of malaria-naive individuals, termed volunteer infection studies (VIS) (9) or controlled human malaria infection (CHMI) studies, provide an opportunity to evaluate antimalarial activity in humans with low parasite burden in a controlled setting. These studies avoid the confounding factors of drug efficacy observed in clinical malaria cases, such as acquired immunity, concomitant diseases, and medication (10–12).

Drug efficacy indices, used to summarize drug effect over time, are key measures by which to evaluate the progression of drug candidates from the preclinical to clinical stages of the drug development pipeline. For malaria, pharmacodynamic/efficacy indices include the following: (i) measurements of total or proportional parasite clearance, such as the parasite clearance rate or parasite reduction ratio over 48 h (PRR_48_) (4); (ii) drug exposure typically reported by indicative drug concentrations, such as the MIC (8); and (iii) clinical endpoints, such as adequate clinical and parasitological response (ACPR) (13). Parasite clearance measures are widely used in antimalarial drug development to guide compound selection (2) and are also reported in clinical studies in endemic areas as a measure of drug efficacy (14).

In a previous paper, we developed a suite of mathematical models of parasite growth and drug-parasite dynamics to investigate murine malaria infection and malaria drug experimental tests. Through extensive simulation of these models and comparison with data for several drugs, we found that the experimental systems and differences between the two murine malaria infections had appreciable effects on measured drug efficacy and treatment outcomes. More specifically, we found that drug efficacy is influenced by host-parasite dynamics in P. berghei-NMRI mouse infection where resource limitation is caused by aggressive parasite growth, namely, limitations of red blood cells (RBCs) (15). In P. falciparum-SCID mouse infection, we found that continued injections of human RBCs have a noticeable impact on subsequent clearance patterns of uninfected and infected RBCs (iRBCs). We additionally identified mechanisms of observed recrudescence patterns after noncurative treatment that are not discernible from the experimental data nor captured by current modeling approaches of antimalarial drugs. These unknown mechanisms may include altered parasite maturation or dormancy and affect experimental measures of cure, thus limiting interpretations of curative dose for a particular drug (15).

In this study, we examined the ability of the parasite clearance after treatment estimated from murine experiments to translate to estimates in human studies. This analysis used data from studies of two antimalarials, MMV048 and OZ439 (artefenomel), in the P. berghei-NMRI mouse, in P. falciparum (strain 3D7)-SCID mouse, and in P. falciparum (strain 3D7)-human infection experiments. Both compounds are part of the Medicines for Malaria Venture portfolio (https://www.mmv.org/research-development/mmv-supported-projects). OZ439 is a synthetic peroxide antimalarial candidate that is fast acting against all asexual erythrocytic parasite stages. It is currently being evaluated in combination with ferroquine in phase II clinical studies (11, 16–18). MMV048 is a *Plasmodial* phosphatidylinositol 4-kinase (PI4K) inhibitor efficacious against liver erythrocytic parasite-life cycle stages currently in phase IIa (19–21).

**FIG 1:**
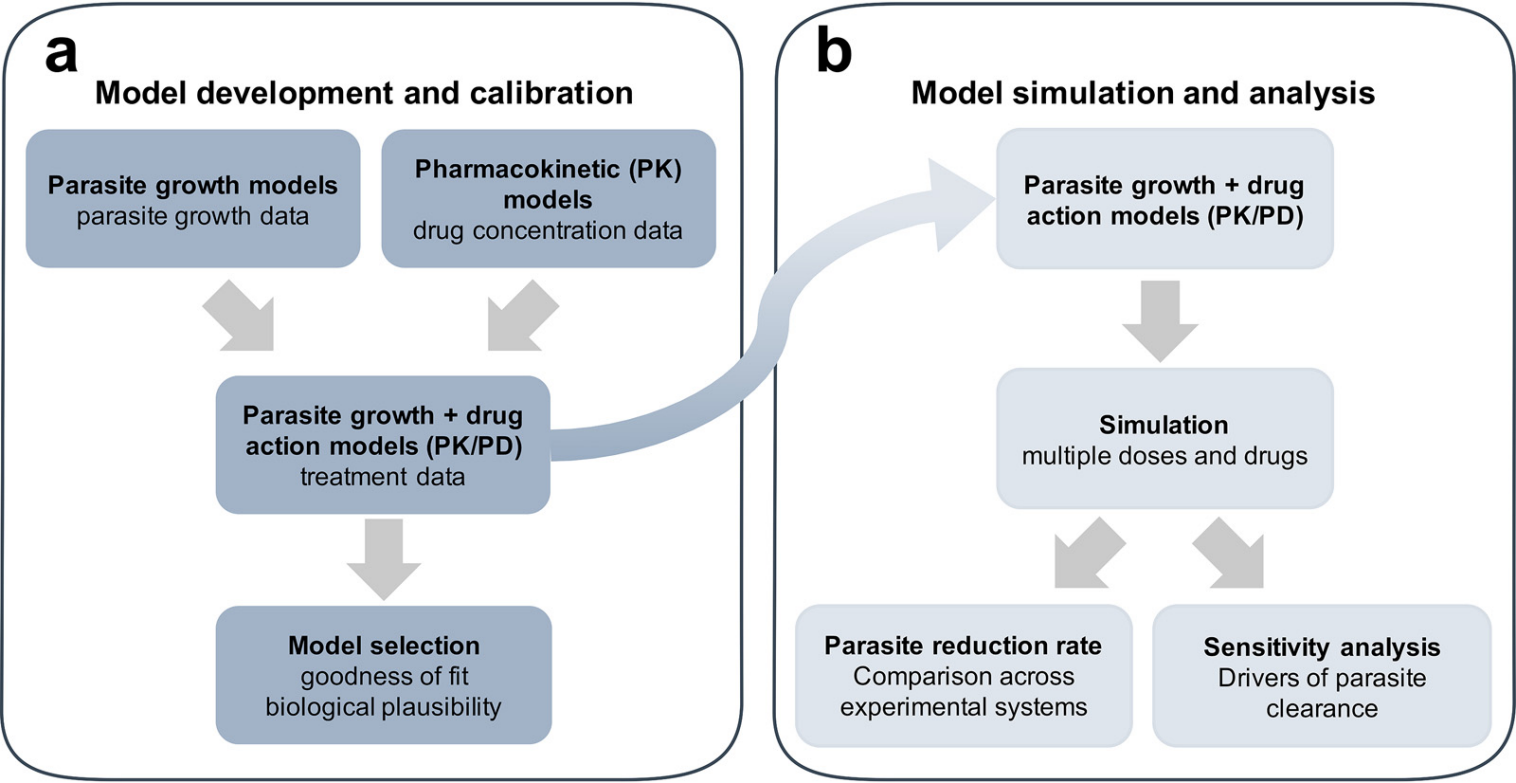
Standardized workflow for the systematic investigation of parasite-host-drug dynamics throughout the (pre)clinical antimalarial development process. (a) Mechanistic models of parasite growth are calibrated to extensive undisturbed parasite growth (control) data in murine and human infection experiments on a population (mouse) or individual (human) level. Combined with models of drug concentration (PK) over time, they are used to calibrate to treatment data over multiple doses and drugs. Models were selected for further analysis based on appropriate goodness-of-fit measures and assessment of biological plausibility. (b) Model simulation over multiple drugs and doses facilitates the comparison of the parasite reduction rate over all experimental systems. Subsequent sensitivity analysis allows the identification of parasite, host, or drug dynamics as the drivers of experimental outcomes.

We modeled parasite growth and clearance following two new antimalarial treatments in murine and human malarial infections (Fig. 1a, model development and calibration). We included relevant and potentially important features of parasite growth, host, and drug dynamics in the models of all three testing systems following a comprehensive examination of their biological and experimental background. Through simulation and global sensitivity analysis of these models, we explored and compared the estimates of parasite clearance for a range of dosing regimens and parasite and host assumptions in all three systems (Fig. 1b). Through this analysis, we demonstrated which of the factors (host, parasite, host-parasite, and drug dynamics) primarily determine the relationship between parasite clearance estimates across the antimalarial drug development pathway and assess implications for decision making in drug development.

## RESULTS

**FIG 2:**
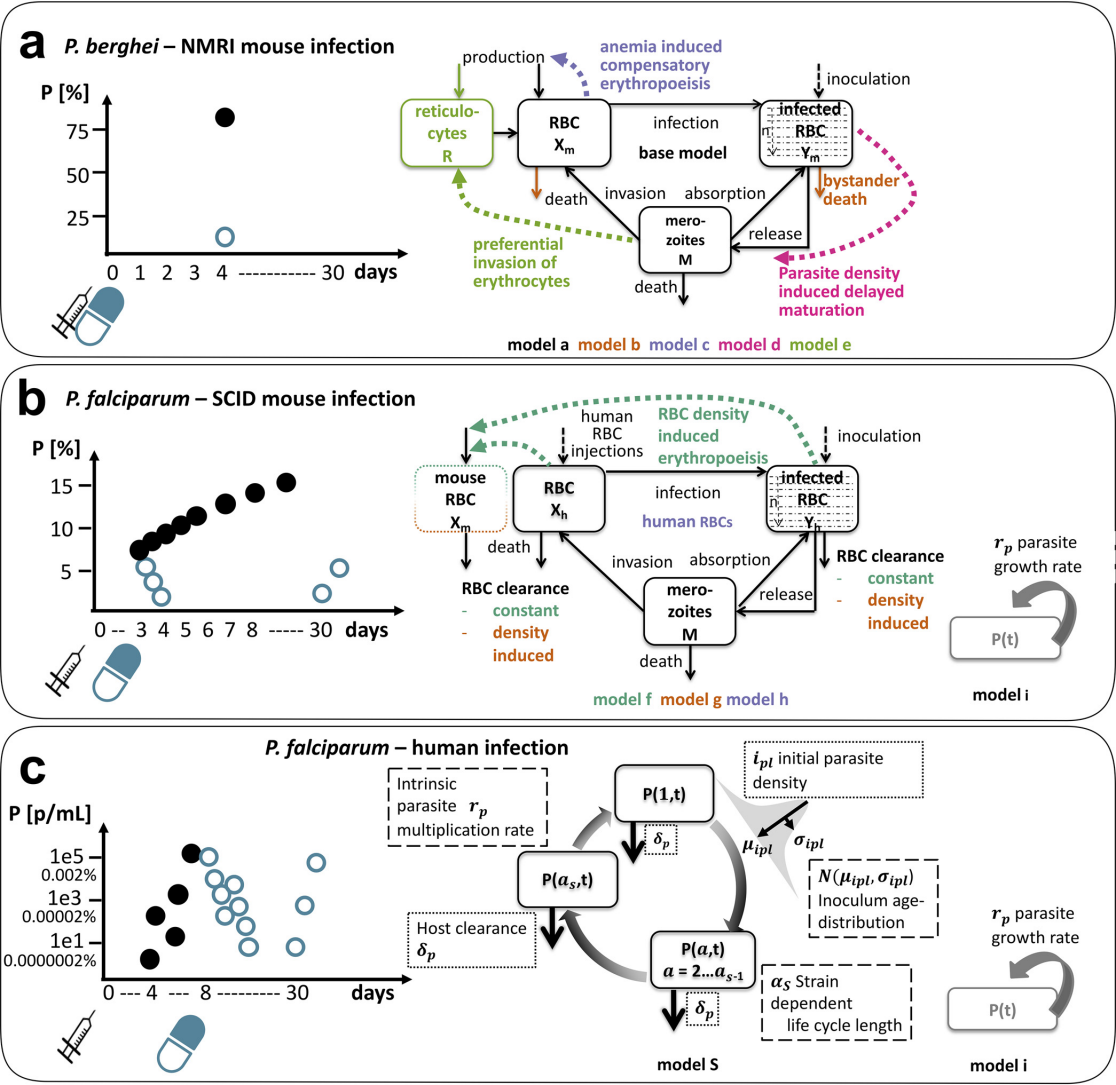
Experimental sampling design and investigated parasite-host dynamics in preclinical and clinical antimalarial drug development. Parasitemia of a typical subject in each experimental system is shown on the left side. Subjects are inoculated on day 0 (black syringe), and treatment (blue syringe, oral dose) commences on the same day or up to 4 days afterward in murine malaria infection (a, b) and after 7 to 9 days in human infection (c). Each dot represents one measurement, with black dots indicating untreated growth/growth before treatment and blue dots representing parasitemia after treatment. Separate control groups were measured in murine experiments. The murine infections are measured in percent of infected RBCs *P* (%) (a, b), and human infections are measured in infected RBCs per milliliter *P* (parasites/ml) (c) (Table 1). For illustrative purposes, we added a conversion of *P* (parasites/ml) to *P* (%) in VIS (c) based on the assumption of 5 × 10^9^ RBCs per milliliter in male humans (22). The schematics of mathematical models used to describe parasite growth in the respective system are shown on the right side. (a, b) In murine malaria infection, capturing uninfected (*X*) and infected (*Y*) RBC dynamics is crucial to understand implications of resource limitation (P. berghei-NMRI infection) and resource replenishment (P. falciparum-SCID infection) (15). Details on murine model structure can be found in Material and Methods (equations 1 to 6). In contrast, low total numbers of parasites *P*(*t*) in P. falciparum-human infection and increased number of measurements shift modeling focus on dynamics of intraerythrocytic parasite stages over time (c). The exponential growth model i can only be used to capture parasite growth of P. falciparum in SCID mouse and human, as data are not informative enough in P. berghei-NMRI infection.

The design of the studies/experiments used to evaluate the candidate antimalarials (MMV048 and OZ439) in normal mice, SCID mice, and human volunteers is described in Table 1 and Fig. 2a to c (left side). Mice were infected with inocula containing around 2 × 10^7^ parasites or more, resulting in progression to severe disease with high parasitemia of up to 60 to 80% (P. berghei, parasitized mouse RBCs) and 15 to 20% (P. falciparum, parasitized human RBCs) within a week of inoculation. By design, human volunteers do not progress to high parasitemia to avoid severe clinical illness (9). Volunteers reach parasitemia of around 10^4^ parasites/ml (corresponding to 0.0002% under the assumption of 5 × 10^9^ RBCs/ml in male humans [22]) before treatment.

**TABLE 1:** Overview of the data and experimental outcomes in our analysis of murine and human malaria infection^*a*^

Parameter	P. berghei ANKA in NMRI mice	P. falciparum (3D7) in SCID mice	P. falciparum (3D7) in human
No. subjects			
Control (separate)	215 (Yes)	132 (Yes)	177 (No)
MMV390048	65	50	20
OZ439	200	48	24
No. subjects/dose	3–10	1–2	6–8
No. dose levels			
MMV390048	7 SD; 5 QD	2 DD; 6 QD	3 SD
OZ439	14 SD; 3 TD	10 SD; 1 DD	3 SD
Minimum expt length (d)	3–4 (up to 30)	8 (up to 31)	8 (up to 30)
Inoculum (no. iRBCs)	2 × 10^7^	3.5 × 10^7^	1,800–2,800 (viable parasites)
Total cure (no. mice)			
MMV048	4 × 3 mg/kg (3)	4 × 20 mg/kg (2)	1 × 80 mg (8)
OZ439	1 × 30 mg/kg (30)	1 × 100 mg/kg (2)	
Outcomes	Activity; cure (survival, parasite clearance)	PKPD relationship; parasite clearance; cure	PKPD relationship; parasite clearance; cure
Parasitemia output	Percentage of infected RBCs	Percentage of infected RBCs	Concn of infected RBCs per ml

a Untreated parasite growth behavior was informed by a separate control group in murine experiments and by parasite growth data before treatment commences in human infection. Experimental outcomes evolve over the preclinical and clinical stages with increasing data richness per subject over time from crude efficacy measures, such as activity and parasite reduction, to more detailed concentration effect relationships. Measures of parasite reduction (e.g., parasite clearance rate or proportional antimalarial activity) are frequently used to evaluate compounds throughout the clinical development stages (2). SD, single dose; DD, double dose; TD, triple dose; QD, quadruple dose.

### Parasite-host dynamics and experimental design influence treatment in murine malaria infection.

In our previous work (15), we developed several mathematical models of parasite and drug dynamics in both P. berghei-NMRI and P. falciparum-SCID mice (Fig. 1a). The experimental system and mathematical models are described in Fig. 2a and b, respectively. Using the parameter estimates from the original paper (for both parasite growth and pharmacokinetic-pharmacodynamic [PKPD] models), we simulated the models of murine infection to compare their results with our VIS simulations below (Fig. 1b). Here, we primarily compared parasite clearance after treatment across the three testing systems (Fig. 1b, comparison of parasite clearance across experimental systems) as estimated from the models for a range of drug doses. We subsequently assessed the influence of experimental design and parasite-host system on parasite clearance throughout the antimalarial development pathway by sampling from the posterior distributions for VIS and by sampling parameters from a log-normal distribution with 20% standard deviation for the murine models (Fig. 1b, sensitivity analysis).

In the two murine systems, several experimental and parasite-host traits were examined by including them in a suite of models (further details in Materials and Methods and Table S5 in the supplemental material) (15). From an experimental perspective, data availability and experimental design differ between the two murine infection systems. For example, in P. berghei-NMRI infection, the volume of blood sample needed for analysis and aggressive parasite growth limits the frequency of collection for measurements of parasitemia. From the host perspective, we investigated and quantified adaptions of the host-system to increasing parasite burden. In P. berghei-NMRI infection, we included erythropoiesis (23, 24) or clearance patterns as well as parasite adaptions, such as parasite maturation (25) and target RBC preferences (23), in our models to capture patterns of RBC availability and the occurrence of anemia (Fig. 2a). In P. falciparum-SCID infection experiments, continued injections of human erythrocytes hinder the occurrence of anemia and lead to an increase in the proportion of available human host-cells throughout the experiment (Fig. 2b). The injected human RBC volume of 4.55 × 10^9^ RBCs every 2 to 3 days (corresponds to 46% of total mouse RBCs) leads to a high clearance of excess erythrocytes throughout the experiments, thus likely affecting measures of drug efficacy (6). Different hypotheses regarding the influences on RBC clearance mechanisms were formalized in our models of P. falciparum-SCID mouse infection (Fig. 2b).

Using a maximum likelihood approach, we estimated the parameters of our parasite growth models by fitting them to the pooled experimental control data of untreated parasite growth. The fitted models were then combined with the pharmacodynamic (PD) models to analyze the influence of the parasite-host dynamics and experimental design on parasite clearance after treatment.

For MMV048, we captured the parasite clearance data after treatment by including a delayed parasite clearance of parasites damaged or killed by the drug, modeled by the clearance rate of dead parasites CL_Y_, in both murine hosts (Table S5, clearance PD model selection, CL_Y_ range of 0.036 to 0.041 [1/h] in P. berghei-NMRI and CL_Y_ range of 0.068 to 0.071 [1/h] in P. falciparum-SCID infection over all mechanistic parasite growth models). In contrast, for OZ439, we observed a delayed drug effect through a turnover (Table S5, turnover PD model selection, *k_R_* (first-order rate constant for biological intermediate) range of 0.013 to 0.06 (1/h) in P. berghei-NMRI and *k_R_* range of 0.013 to 0.016 (1/h) in P. falciparum-SCID infection over all mechanistic parasite growth models) (15). In both murine experimental systems, we found that assumptions on parasite-host dynamics result in differing estimates of parasite clearance times, therefore influencing the evaluation of new compounds (see Fig. 4 in reference 15).

### Human volunteer infection studies exhibit large variation in observed parasite growth and clearance dynamics.

We modeled parasite growth prior to treatment using data from 177 volunteers in 27 treatment cohorts in VIS conducted at Berghofer QIMR, Brisbane between 2012 and 2016 (26). These cohorts were part of 14 studies investigating new antimalarials in development and currently available antimalarials. Volunteers were infected with 1,800 to 2,800 infected RBCs and treated 7 to 9 days after inoculation (26).

Sampling frequency of human volunteers was much higher compared with that of murine experiments; however, the number of measurements above the lower limit of quantification (LLOQ) (111 parasites/ml) varied between cohorts and volunteers with a median of five quantifiable measurements (ranging from one to eight). These detailed data allowed us to capture features of the parasite life cycle, such as the characteristic oscillation in parasite densities. This phenomenon is caused by periodical sequestration of late-stage asexual parasite stages to the deep microvasculature of the host organs (27) and synchronicity in parasite growth determined by the distribution of parasite age in the inoculum (28).

The models of asexual parasite growth in human volunteers are described in Fig. 2c. We adapted a discrete time model (29) previously used to investigate antimalarial treatment (28), resistance against artemisinin combination therapy (30), and the impact of new antimalarial combinations (31). A set of difference equations (model S parasite stages, equation 7) describes the parasite inoculation, capturing its size and age distribution, and the subsequent mechanism of intraerythrocytic parasite development including ageing and parasite death. Based on analysis by Wockner et al., that quantified parasite growth behavior of P. falciparum (3D7) in VIS, the life cycle length was set to 39 h (26). For comparison, we tested a second model that assumes exponential parasite growth (model i, equation 10), summarizing parasite replication and death into one growth parameter. This model is commonly used to capture parasite growth and treatment effects in antimalarial drug development (8, 11).

**FIG 3:**
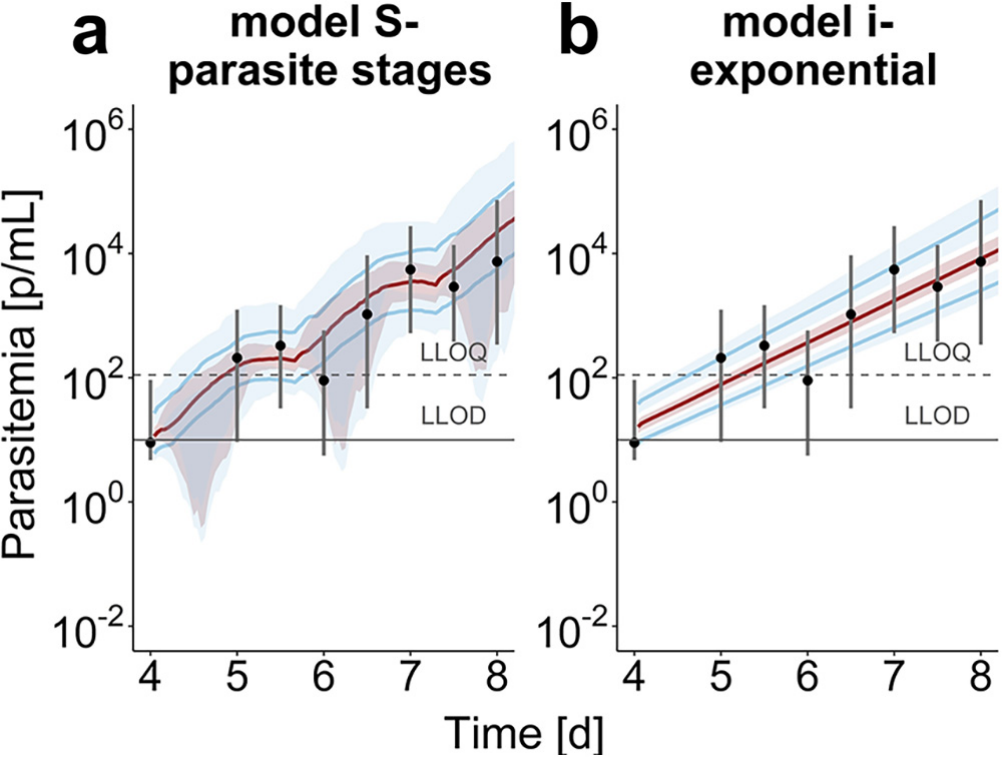
Population parasite growth prediction in VIS. Median parasite densities (black dots) with their 90th percentiles over the time starting 4 days after inoculation for the 177 subjects in VIS (26). Model predictions show the median (red) and 90th percentile (blue) with credible intervals over 100 trials with 20 subjects. (a) The mechanistic growth model S captures parasite growth trends well over time with discrepancies between data and prediction being centered around parasitemia under the lower limit of quantification (LLOQ) of 111 parasites/ml and lower limit of detection (LLOD) of 10 parasites/ml. We found a population posterior median (credible interval) of the initial parasite load (4 days after infection) of 2.59 (2.44 to 2.74) [log(parasites/ml)], a median parasite age μ_ipl_ of 14.0 h (12.1 to 15.6) with a standard deviation σ_ipl_ of 4.32 h (3.83 to 4.90). The intrinsic parasite multiplication rate *r_p_* of 55.2 (46.3 to 68.5) and death rate δ*_p_* of 0.0302 (0.0263 to 0.0353) (1/h) describe the intraerythrocytic replication dynamics of the parasite. (b) The exponential parasite growth in model i leads to linear growth behavior on the log-scale and so does not capture the oscillating parasite growth behavior. We estimated a growth rate *r_p_* of 0.0649 (1/h) (0.0620 to 0.0678). The posterior predictive checks are illustrated in Fig. S7 (model S) and Fig. S8 (model i) in the supplemental material.

We incorporated the models into a Bayesian hierarchical framework to estimate the posterior distributions of the model parameters from the VIS data. We investigated different levels of parameter variability, distinguishing between parasite and host-dependent parameters by varying hierarchical parameter allocation in different parasite growth model specifications (see Table S2, models S1 to S3, in the supplemental material). Model S3, from here on referred to as model S, was selected based on the Watanabe-Akaike information criterion (WAIC) (Table S2). The parameters describing distribution of the initial parasite load (mean μ_ipl_ and standard deviation σ_ipl_) and the intrinsic parasite multiplication factor, *r_p_*, were found to vary on a cohort level, whereas the initial parasite load *i*_pl_ and parasite death rate due to host clearance δ*_p_* vary between individuals (see Fig. S7 in the supplemental material). The population posterior predictions of the two parasite growth models (Fig. 3) begin on day 4 after inoculation when quantifiable parasitemia measurements from the volunteers are first available (Fig. 2). We estimated a parasite multiplication factor pmf of 17.0 (15.3 to 20.0) over one life cycle of 39 h (equation 7). Identifiability issues of growth rate *r_p_* were detected by the partial congruency of the marginal prior and posterior distributions (Fig. S7). This means that data on parasite growth are not informative enough for estimating this parameter, similar to previous model analysis (32). For exponential growth model i, we estimated a pmf of 12.61 (11.2 to 14.2) over one parasite life cycle. Differences in the parasite growth rate *r_p_* between individuals within cohorts could not be linked to cohort or subject-specific parameters for model i (see Fig. S8 in the supplemental material).

Given our calibrated VIS parasite growth models, we incorporated PK models and modeled drug effects (pharmacodynamics) (Fig. 1a) to analyze single-dose treatment of 20 volunteers with MMV048 and 24 volunteers with OZ439 (see Table S1 in the supplemental material). Drug concentrations after treatment were predicted using pharmacokinetic (PK) models (equation S1and S2 in the supplemental material) and individual PK parameters specified (Data Set S1 in the supplemental material). We simulated individual PK profiles to exclude large variation in drug concentration as an influencing factor in the following analysis. Drug concentration over time was described by a two-compartment model with zero-order absorption for MMV048 and a 2-compartment model with linear absorption for OZ439. In the following section, we explain the results for OZ439. The analysis of MMV048 is detailed in the supplemental material, and any deviations are highlighted and discussed here.

We incorporated a direct effect of drug concentration on parasite death (equation 8) and additionally tested for drug concentration-induced retarded parasite growth (equation 9) due to the reduced parasite growth observed after noncurative dosing with OZ439 and the shift in oscillation patterns to a longer period. This phenomenon was also previously described for artemisinin derivatives (33). A comparison of drug action model based on WAIC and additional observations are included in Table S3 in the supplemental material.

We detected drug concentration-dependent prolongation of parasite stages after treatment with MMV048 and OZ439, meaning that at sufficiently high drug concentrations, each life cycle stage can be prolonged by 26 min or 65 min, respectively (equation 13). Through description of individual treatment effects via subject-specific parameters of drug efficacy (see Fig. S9 to S12 in the supplemental material), we could capture individual parasite clearance and recrudescence curves after treatment with MMV048 (see Fig. S3 and S4 in the supplemental material) and OZ439 (see Fig. S5 and S6 in the supplemental material) for all subjects.

**FIG 4:**
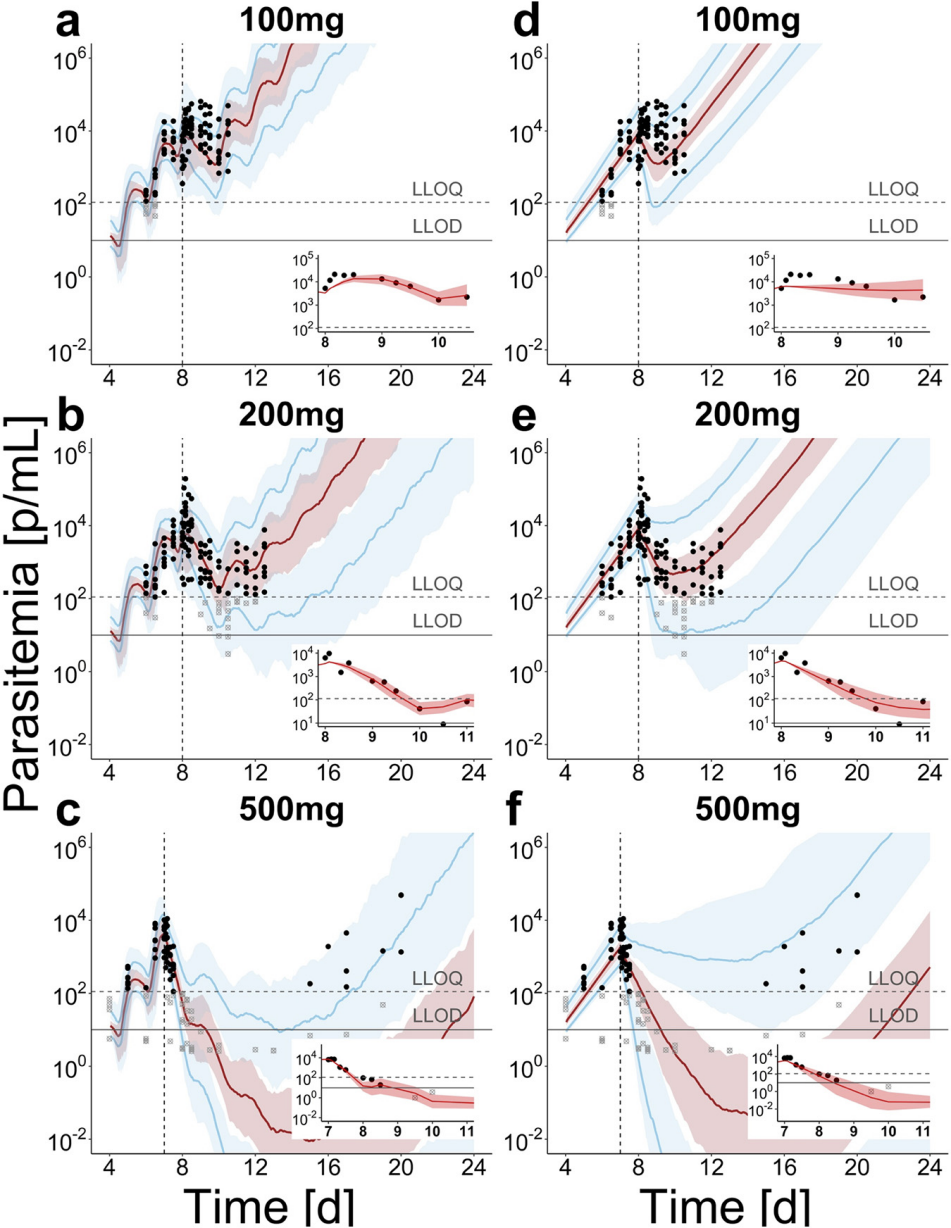
Population prediction after treatment with OZ439 in P. falciparum-human infection for mechanistic growth model S (a to c) and exponential model i (d to f). The simulated median (red) and 90th percentile (blue) with credible intervals over 100 trials with 20 subjects are compared to data of individual parasite densities (black dots) in the respective treatment group. For each treatment group, parasite clearance of a typical subject (subjects 1, 15, and 19) immediately after treatment is illustrated with individual predictions in the inset figures (for all subjects, see Fig. S12 and Fig. S13 in the supplemental material). Immediately after treatment with 100 mg OZ439 (a, d) an increase in parasite densities and transient decelerated parasite growth was observed, which is captured by model S through the lengthening of the parasite life cycle. After treatment with higher doses (b, c; e, f), this effect is less influential, with more prominent parasite killing by the drug. After treatment with 500 mg (c, f) only four out of eight volunteers exhibited parasite recrudescence (see individual data in Fig. S5 and Fig. S6 and posterior distributions in Fig. S11 and S12 in the supplemental material). Vertical line indicates time of treatment, and the horizontal lines indicate the LLOQ of 111 parasites/ml and LLOD of 10 parasites/ml.

Although the posterior predictions of recrudescence (Fig. 4) fit the data well, we note that informed mechanistic models and inclusion of experimental recrudescence outcomes and cure are hindered by limited data on cure probability since each cohort only consisted of eight subjects. Additionally, insights into minimum parasite concentrations for cure are missing.

### Parasite clearance is unlikely comparable across the preclinical and clinical development pathway.

The parasite clearance after treatment was estimated from our model simulations for murine and human malaria infection (Fig. 1b) over a wide range of single doses of each drug. The range of doses and models facilitated comparison across preclinical and clinical stages. We chose seven doses per experimental system that capture realistic testing dose ranges. We simulated 1,000 trials for each murine experiment, with parameter estimates published in reference 15 (see Table S5 for an overview) and variability added between trials, with parameters fixed within one trial over all doses. In human-P. falciparum infections, we simulated 100 trials with 20 subjects per dose and varied the parasite-dependent parameters between trials and the host-dependent parameters between subjects within a trial. The parameters of the PK models were fixed to population values for all model simulations to allow for adequate characterization of host-parasite-dependent dynamics and variability. The simulation setup is detailed in Table S4 in the supplemental material.

**FIG 5:**
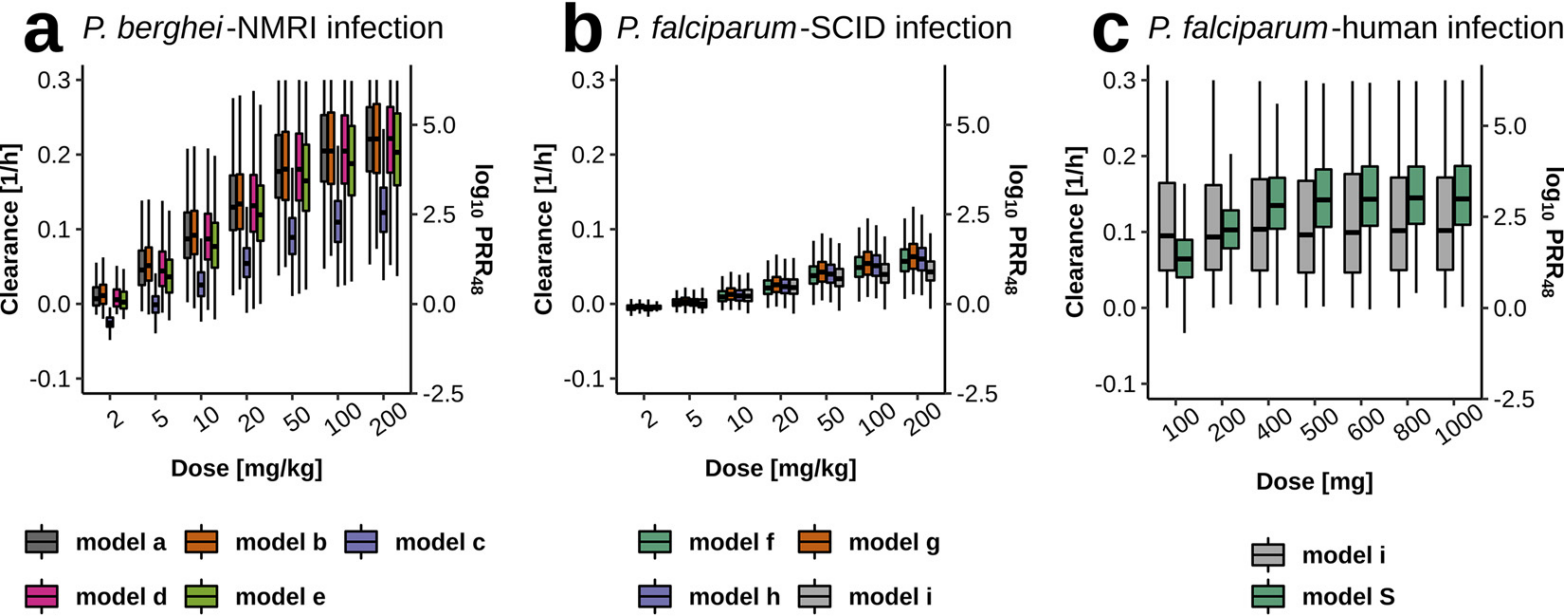
Parasite clearance across the murine and human experiments after single-dose treatment with OZ439. Within one experimental system, the predicted clearance rates are fairly consistent. (a) Model c (compensatory erythropoeisis) of P. berghei-NMRI infection estimated a lower maximum drug effect *E*_max_ resulting in lower clearance rates (see Table S5 in the supplemental material). (b) Compared to panels a and c, P. falciparum-SCID infection shows less variability. (c) The wide clearance range observed in model predictions of the exponential growth model i for P. falciparum-human infection stems from the wide posterior distribution of the maximum drug effect *E*_max_. Parasite clearance rates were calculated from simulation output using the methodology provided previously (49). For comparability, a conversion to log_10_ PRR_48_ was added as a secondary *y* axis where log_10_ PRR_48_ = log_10_(*e*^parasite clearance rate ×48^).

Figure 5 compares parasite clearance calculated from simulation output after single-dose treatment of OZ439 over the two murine and experimental human infection experiments. We find varying relationships of estimated parasite clearance with doses across the experimental systems. Median estimates of parasite clearance rates plateau around 0.2 (1/h) (log_10_ PRR_48_, 4.17) in P. berghei-NMRI infection and 0.05 (1/h) (log_10_ PRR_48_, 1.04) for P. falciparum-SCID infection. Analysis for MMV048 shows similar maximum clearance rates in murine infection (see Fig. S13 in the supplemental material) and varying relationships with dose. In experimental P. falciparum-human infection, the parasite clearance rate for OZ439 plateaus with model S around 0.12 (1/h) (log_10_ PRR_48_, 2.50). The ranges of the predicted clearance time of P. falciparum-human infection between model i and model S are also reflected in the wider posterior predictive intervals of the former after treatment with OZ439 (Fig. 4).

Parasite clearance is influenced by varying parasite-host dynamics throughout the analyzed experiments of murine and human malaria infection. Despite the poor comparability across experimental systems, the parasite clearance predicted from mechanistic models within an experimental system are comparable but with different levels of variation. Via a global sensitivity analysis, we thus identified which host, parasite, and drug dynamics cause the highest variability in parasite clearance after treatment with MMV048 and OZ439. We first partitioned variability by classifying parameters by their influence and dependency on parasite, host, or drug concentrations. For example, the parameters of infection-induced RBC clearance γ in murine malaria models are classified as host-parasite parameters since they are induced by parasite concentration and influence overall RBC concentration (Table 2). We then undertook a global sensitivity analysis of the models on simulated parasite clearance rates, reducing computational time by using emulators and Sobol analysis, which calculates first-order indices and total effect indices to evaluate individual and combined parameter contributions to the variance of parasite clearance rates (see Materials and Methods).

**TABLE 2:** Classification of model parameters into host, parasite, host-parasite, and drug parameters^*a*^

Parameter	Model parameter(s)		
	P. berghei-NMRI	P. falciparum-*SCID*	P. falciparum-human
Host		Initial percentage of human RBCs *H*_0_; base death rate of all RBCs λ; Parameters of RBC density dependent RBC clearance χ_max_ and *k*χ_50_	Parasite death rate at each time stage δ*_p_*
Parasite	Infectivity parameter β; no. of merozoites per infected RBC *r*; attraction of parasite to reticulocyte ε; parasite inoculum viability ω	Infectivity parameter β; no. of merozoites per infected RBC *r*; parasite inoculum viability ω; expt parasite growth rate *r_p_*; initial parasitemia *P*_0_	Intrinsic parasite multiplication rate *r_p_*; distribution parameters of the initial parasite density μ_ipl_ and σ_ipl_
Host- parasite	Parameters of infection-induced RBC clearance γ_max_ and *k*γ_50_; conc. of infected mouse RBCs achieving 0.5 growth retardation effect *k*_l,50_	Parameters of infection-induced RBC clearance γ_max_ and *k*γ_50_; base clearance of infected RBCs φ	Initial parasite density *i*_pl_
Drug	Parameters of concn effect relationship *E*_max_, EC_50_; clearance rate for damaged parasites CL*_Y_*; first-order rate constant for biological intermediate *k_R_*	Parameters of concn effect relationship *E*_max_, EC_50_; clearance rate for damaged parasites CL*_Y_*; first-order rate constant for biological intermediate *k_R_*	Parameters of concn effect relationship *E*_max_, EC_50_; growth retardation parameter *k*_ret_

a Parameters were classified based on their dependency on host and parasite variable states and induction through those variables. Detailed description of parameters for murine models of P. berghei-NMRI and P. falciparum-SCID infection can be found in reference 15. Details on parameters of P. falciparum-human infection can be found in Fig. 2 and Table 3.

**FIG 6:**
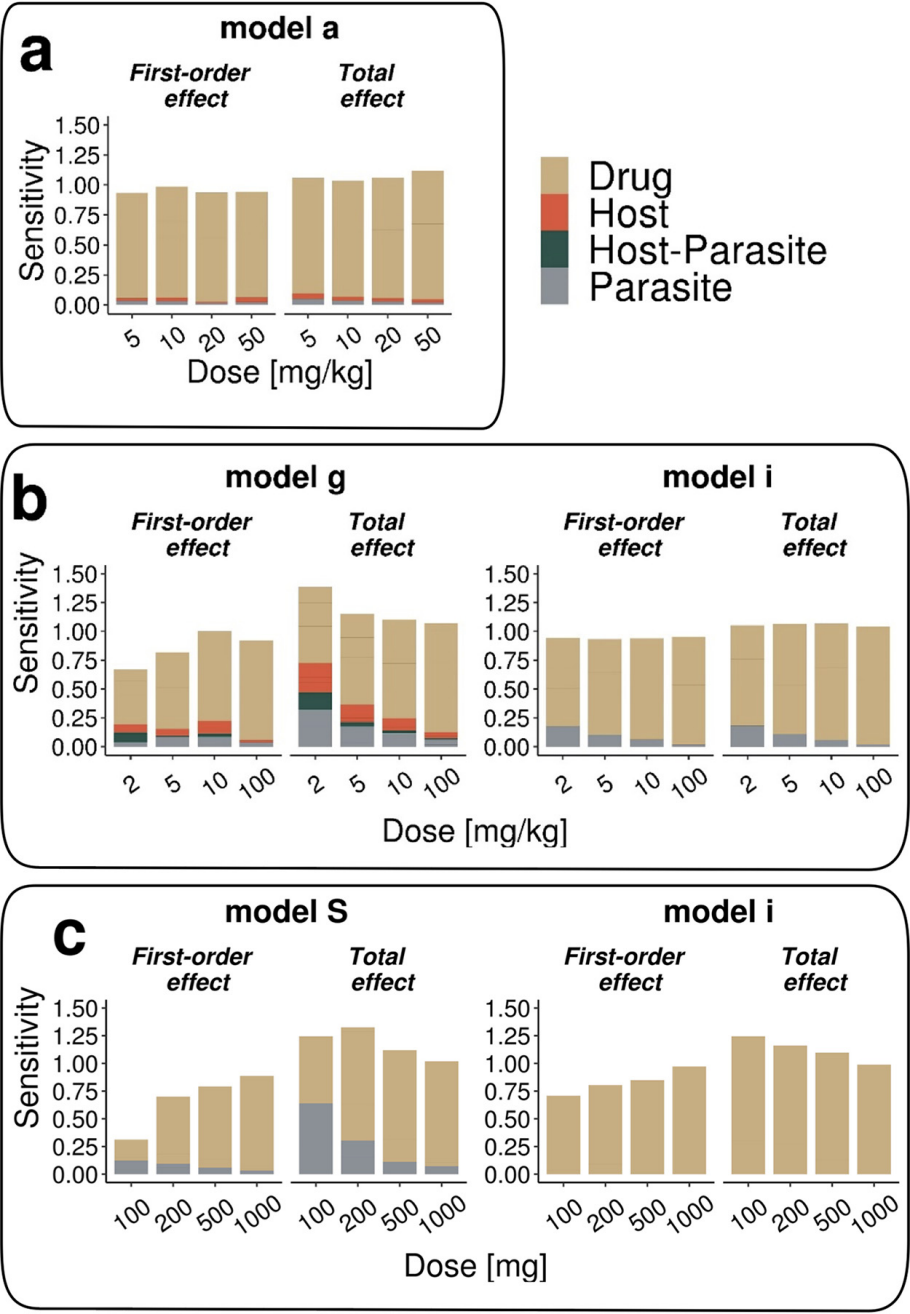
Exemplary results of the sensitivity analysis of parasite clearance after OZ439 treatment toward parameters of host, parasite, host-parasite, and drug dynamics for P. berghei-NMRI (a), P. falciparum-SCID (b), and P. falciparum-human infection (c). Using Sobol sensitivity analysis, first-order effects measure individual parameter contributions, and the total effect indices summarize individual and interactive parameter contributions to the outcome variance (see Materials and Methods).

In general, we find in all experimental systems that parasite clearance after treatment is most sensitive to drug action parameters (see Table 2). This is expected, as we are measuring the effect of the drug in these systems. We also find that interactions of the drug action parameters (increased total effect over first-order effect) occur over all doses but decrease with increasing dose, evident in the convergence of first-order and total effect values. However, in both the murine (Fig. 6b) and human P. falciparum (Fig. 6c) infections, we find at lower doses an increasing influence of both the host or parasite or host-parasite parameters. This indicates a greater influence of the experimental system on measured parasite clearance. Even in medium-high dose ranges, drug-unrelated parameters and their interactions account for up 25% of variance in parasite clearance. This is mainly caused by sensitivity toward maximum infection-induced RBC clearance γ_max_ and parameter φ, which capture splenic/liver clearance, and maximum infection-induced RBC clearance γ_max_. The exponential growth model i shows diminishing dependence on parasite replication rate *r_p_* with increasing doses in P. falciparum-SCID infection.

Estimated treatment effects of experimental P. falciparum-human infection are sensitive to the maximum drug action parameter *E*_max_, resulting in a large range of parasite clearance rate prediction throughout all doses. In the lower dose ranges of OZ439 treatment, the total effects of the age distribution parameters μ_ipl_ and σ_ipl_ of model S account for up to 50% of all variance (Fig. 6c). In contrast, throughout all regimens, parasite parameters have negligible influence on clearance for MMV048 (see Fig. S14 in the supplemental material). The full set of individual parameter contributions in all experimental systems can be found in Supplemental Data Set S2. The complete set of results for both drugs is shown in Fig. S14 and Fig. S15 in the supplemental material.

## DISCUSSION

Our analysis of data from three unique experimental systems of drug action in *Plasmodium* infection, P. berghei-NMRI mouse infection, P. falciparum-SCID mouse infection, and the human VIS, demonstrates a discrepancy in influencing parasite, host, and drug dynamics on parasite clearance between the preclinical and clinical antimalarial testing stages. This complicates the translation of results between these different experimental systems, which aim to collectively inform drug development. We initially intended to identify antimalarial drug efficacy indices that were reliable for translation. Although our analysis did not result in a unifying output, our insights into parasite-host dynamics across the preclinical murine and early clinical experimental infections in humans via the mathematical models provide a pathway to facilitate inference from the data. By describing the different experimental testing systems with data-calibrated mechanistic models, we used model predictions coupled with sensitivity analysis to identify factors significantly influencing experiments aimed to characterize drug efficacy. In addition to the drug and dose being tested, we identified differing magnitudes of host-parasite dynamics between the experimental systems as a driver of estimated parasite clearance rate.

By employing mechanistic models of parasite growth and drug effects in all three testing systems, we found that several important dynamics affect translation of parasite clearance after treatment and likely other parasitological outcome measures between preclinical and clinical systems.

Firstly, as expected, our sensitivity analysis of parasite clearance revealed a high sensitivity to parameters of drug action in higher dose ranges in all three experimental systems. In contrast, we found a variation in overall sensitivity of parasite clearance to parasite-host dynamics in lower-medium dose ranges between the three experimental systems. In murine and human P. falciparum infection, parameters of parasite growth influenced parasite clearance after treatment with lower doses. Additionally, the occurrence of clearance mechanisms of excess RBCs in P. falciparum-SCID infection could place additional constraints on parasite growth. Overall, the increased susceptibility to the experimental setup of the SCID system limits direct translation and comparison of results between laboratories and highlights the need for strict experimental protocols (15, 34). Thus, to predict human equivalent doses by exploiting the value of the P. falciparum-SCID mouse system, additional information is required concerning the interplay of disease dynamics and the experimental background, which could be quantified with our modeling approach. For P. berghei-NMRI infection data, estimation of the parasite clearance was less sensitive to parasite-host dynamics. However, the aggressive parasite growth in this experimental system might lead to host-cell limitations and, therefore, different growth dynamics compared to P. falciparum infection, challenging the translatability of drug efficacy measures. Although not investigated here, the uncertainties regarding clearance mechanisms may also hinder the investigation of drug-drug interactions. The testing of combination therapies relies on the ability to allocate contribution of each drug to effects on parasite clearance and to estimate potential drug interactions. *In vitro* experiments could prove to be valuable alternatives in establishing formal descriptions of these interactions, which could thus be incorporated into mathematical models to predict the efficacy of combination (31).

Secondly, the sensitivity of parasite clearance measured at lower drug doses due to factors other than the drug raises questions for current approaches to determine concentration-effect relationships (equation 12) that rely on information gained in lower-medium dose ranges to define the concentration with half-maximum effect (EC_50_). Additionally, other common drug efficacy indices, such as the MIC are influenced by EC_50_ measures (equation 17). We, therefore, recommend that inferences based on data gathered at low-medium doses across the preclinical and clinical stages of drug development are assessed by considering factors connected to experimental and host-parasite pairing. For example, in P. falciparum-SCID and P. falciparum-human infection experiments, the sensitivity of the dose-response relationship of data obtained at lower dose ranges should be investigated for each drug candidate to avoid bias in decision making. In experiments with a new compound where drug efficacy analysis reveals a substantial influence of parasite-host dynamics in lower dose ranges, these data should not be used to characterize the MIC for decision making.

Thirdly, our earlier work indicated that recrudescence and thus estimates of MIC are potentially influenced by additional parasite mechanisms, including retarded parasite growth in P. falciparum-SCID experiments (15). Experiments explicitly aimed at observing recrudescence are considered highly informative in understanding the dose-response relationships in these experimental systems. However, utilizing these experiments for modeling proved that estimation or prediction of cure might be hindered by insufficient data on cure rates and curative doses in both murine and human malaria infection (Table 1). Thus, any analysis that we could undertake on cure or recrudescence dynamics is driven by model assumptions and was therefore excluded from this analysis. We further excluded drug concentration-dependent indices, such as maximum concentration, time above a threshold concentration, and the area under the concentration time curve (AUC), because information on PK variability was missing, especially in murine experiments.

How well the parasite clearance rate could be quantified varied between the murine and human volunteer experimental infection data. In the murine malaria experiments, there is limited repeated blood sampling of each mouse, and evaluating progression of infection was limited to measuring percentage of infected RBCs (Fig. 2). Additionally, the evidence for delayed clearance of dead parasites after treatment with MMV048 for both murine models (15) indicates that not only drug properties but also the host’s ability to clear damaged/dead parasites are factors that are determining maximum clearance rate. In the VIS studies, drug treatment is typically administered at an earlier time point at a lower parasitemia, when volunteers are typically asymptomatic. Thus, data from these studies are collected at parasitemia levels up to five logs below those studied in endemic settings, a factor that may confound analysis of maximal parasite clearance.

We were able to compare drug efficacy as parasite clearance across the murine and human infection experiments, thereby measuring drug efficacy throughout antimalarial drug development, because the models formally considered experimental background (i.e., parasite-host combination and experimental procedures) as well as parasite dynamics. Across the mathematical models of parasite growth and treatment, we found that different levels of detail between the preclinical and clinical infection experiments were required to capture parasite growth and thus drug effect. In the mechanistic models of murine malaria infection, high-parasitemia and RBC clearance mechanisms necessitate inclusion of host-cell dynamics in the models. Parasite-host dynamics were influenced by either rapidly increasing parasite load (in the case of P. berghei-NMRI infection) or the experimental setup, including the continued human erythrocyte injections (in the case of P. falciparum-SCID infection). However, in VIS, explicit description of host-cell dynamics is not required due to lower parasite load and relatively short infection follow-up. This means that, as patients are treated due to safety reasons when parasitemia reaches a certain level, only few parasitemia measurements during the initial growth phase are available and any effect of immune response on parasite dynamics is likely to be minimal, thus obviating the need to consider host responses in this model. Furthermore, due to the significant variability in parasite growth observed in the murine experiments, the parasite growth models required calibrated parasite fitness parameters to capture differences between laboratories as well as unmeasured differences in virulence of parasites transferred from donor mice in each experiment (ability of the parasite to infect new RBCs). Similarly, in VIS, parasite growth and synchronicity were dependent on the distribution of viable parasite life cycle stages at inoculation, and models required estimates of these age distribution to recover observed oscillations.

For drug dynamics, we evaluated different pharmacodynamic models to capture the observed parasite clearance in murine and human infection. In murine experiments, delayed clearance of parasites damaged/killed by the drug and drug action through a turnover model were identified. Although *in vitro* experiments reported slight stage specificity of OZ439 (16), with an increased action against trophozoites and schizonts, data were too sparse in the VIS studies to provide a detailed analysis. We did not find evidence of delayed clearance of damaged or dead parasites (27) for the two compounds analyzed. However, this could change for other antimalarials depending on the mode of action, parasite load, and data resolution.

Our estimated value of 17.0 (95% credible interval, 15.3 to 20.0) for the multiplication factor of the parasite after every 39-h life cycle in P. falciparum (3D7) VIS is comparable to previous estimates of 16.4 (95% confidence interval, 15.1 to 17.8) (26) and 21.8 (95% credible interval, 17.6 to 26.9 at a life cycle length of 42 h) (32) in VIS studies. The growth of parasitemia has been modeled using a variety of methods, e.g., testing statistical models for quantification (26), assays for *in vivo* determination (35), and linking it to disease severity (36). However, we found a potential issue of statistical parameter identifiability for this rate where the intrinsic parasite multiplication rate exhibits weak identifiability and strong correlation with the parasite death rate. Nevertheless, we decided to maintain both dynamics, an intrinsic growth rate and parasite death rate, in the model instead of a net growth rate to capture differences between cohorts and individuals and to highlight gaps in knowledge. In theory, the synchronous parasite growth in VIS could inform these two parameters if information on parasite age distributions throughout the parasite life cycle were collected (37). However, this information was not available in the cohorts analyzed and in practice may be difficult to obtain. As parasite numbers after treatment are very low in VIS, the role of immunity for successful treatment is unknown from these studies. However, further analysis and modeling could combine information on parasite growth in clinical field trials in preexposed patients (38–40) and, thus, potentially estimate the effect of the immune system on parasite growth to inform dosing recommendation.

The necessity for timely decisions regarding the progression of antimalarial compounds to the next experimental stages and design of future experiments might not allow for extensive analysis of data through mechanistic models for each drug. However, the mechanistic models developed and validated in the context of this study could serve as a tool to assess potential influences stemming from parasite-host dynamics and help to improve the experimental design of future experiments, providing more depth than the analysis of experimental data alone.

Although drug efficacy data from these three discussed experimental systems allow the comparison of compounds within one *in vivo* experimental system, we are currently not able to translate parasite clearance measures between the systems. Furthermore, as assumptions on the mode of drug action potentially influence clearance patterns, either by data analysis or as predicted by the models, the comparison of compounds with different modes of action is complicated. Our analysis shows that there are different underlying parasite-host dynamics in each system that influence experimental analysis and thus drug efficacy evaluation (either by extrapolation or by models). Therefore, a reliable strategy to translate efficacy measures and produce insights from drug-parasite-host dynamics between the development stages has not yet been identified. Additionally, preclinical and early clinical testing of new compounds is expensive and time-consuming. This is especially true for antimalarials, as only combination therapies will be considered for phase 3 clinical trials and future implementation. To identify appropriate combination therapies, combinations are currently tested in both P. falciparum-SCID experiments (41) and P. falciparum-human infection in VIS (42). Therefore, we suggest that the value of current and new approaches in *in vitro* drug efficacy experiments should be revisited in antimalarial development.

*In vitro* experiments allow the possible identification of mode of drug action, stage specificity, and evaluation of parasite killing in dependence on the unbound drug-concentration without the influence of parasite-host dynamics. This can be achieved with current *in vitro* experiments (43, 44) or by developing new *in vitro* approaches.

Modeling and simulation strategies could thus be informed by more appropriate *in vitro* data unlike today, where large assumptions need to be made on parasite-host dynamics *in vivo*. The *in vitro* dose-response relationship could then be translated to predict human drug efficacy by taking into account parasite growth and clearance mechanisms in human VIS using mechanistic within-host parasite growth models. If validated, this approach could accelerate drug selection in antimalarial drug development by allowing a streamlined prediction of drug efficacy from *in vitro* experiments to humans, therefore reducing the need for substantial or complicated murine efficacy experiments. An investment in setting up new and appropriate *in vitro* experiments and analysis and/or model frameworks could, therefore, support the cost-efficient translation between experimental systems through efficient candidate selection and ability to inform early clinical dosing in real-life settings.

## MATERIALS AND METHODS

### Data, parasite growth, and PKPD models in murine experiments.

Models of murine malaria infection (Fig. 2) were calibrated to extensive parasite growth data and subsequent treatment with multiple antimalarial drugs (Table 1). Here, we briefly present the base structure (model a) of the ordinary differential equations for the murine malaria infection model as specified in reference 15. Parasite growth within the host is described by ordinary differential equations capturing dynamics of uninfected murine host RBCs, *X_m_*_(murine)_; infected RBCs, *Y_Xm_*; and merozoites, *M*. RBC dynamics are incorporated with constant production υ (cells/h) and decay rate μ*_X_m__* (1/h) and become infected through invasion with merozoites dependent on the infectivity parameter β (cells/ml h).

(1) $$\frac{dX_{m}}{\mathrm{dt}}=\nu -{\text{μ}}_{X_{m}}X_{m}-\text{β}X_{m}M$$

Infected RBCs (*Y*) burst after 1/α hours to release *r* new merozoites (*M*) that die with rate δ [1/h]. The aging of the parasite throughout the parasite-life cycle is incorporated via *n* transit compartments.

(2a) $$\frac{DY_{\mathrm{Xm},1}}{\mathrm{dt}}=\text{β}X_{m}M-\text{α}Y_{\mathrm{Xm},1}$$

(2b) $$\frac{dY_{\mathrm{Xm},i}}{\mathrm{dt}}=\text{α}Y_{\mathrm{Xm},i-1}-\text{α}Y_{\mathrm{Xm},I},i=2,\ldots ,n$$

(3) $$\frac{\mathrm{dM}}{\mathrm{dt}}=-\text{β}\left(X_{m}+Y_{\mathrm{Xm}}\right)M+ \alpha rY_{\mathrm{Xm},n}- \delta M$$

The parasitemia, as percentage of infected RBCs, is calculated by summing over the parasite age stages and dividing by the total number of RBCs, as follows:

(4) $$Y_{\mathrm{Xm}}=\sum_{i=1}^{n}Y_{\mathrm{Xm},i}$$

(5) $$P=\frac{Y_{\mathrm{Xm}}}{X_{m}+Y_{\mathrm{Xm}}}100$$

The initial number of infected RBCs is informed by the inoculum size, its viability ω, and the mouse blood volume *V_n_*.

(6) $$Y_{\mathrm{Xm},0,i}=\text{ω}\frac{\text{inoculum}}{V_{n}}$$

For reference, population models for murine parasite growth and drug treatment were estimated via a maximum likelihood approach on trust region optimization.

### Data, parasite growth, and PKPD models in human VIS experiments. (i) Data in VIS.

Parameters for the parasite growth models were estimated using previously published (26) parasite growth data from 177 malaria-naive healthy volunteers enrolled in 27 cohorts as part of 14 VIS studies. Briefly, volunteers were inoculated intravenously on day 0 with human erythrocytes infected with P. falciparum (3D7 strain). Treatment commenced on day 7 to 9 after infection. Parasite growth was monitored using a quantitative PCR assay (P. falciparum 18S rRNA gene). Specifics of parasite growth monitoring and data processing can be found in reference 26.

Parameters for drug efficacy were estimated from parasite clearance data (see Table S1 in the supplemental material) collected from volunteers administered single doses of OZ439 (artefenomel) (11) (cohorts 4, 5, and 6) and MMV048 (cohorts 15, 16, and 27) (20). In the MMV048 cohorts, gametocyte concentration data were also available, where parasite measurements were discarded if the gametocyte count exceeded 10% of the total parasite count. Further details of the data and clinical trial identifiers are given in Table S1. All data was previously published (11, 21). As previously reported, all studies were approved by the QIMR-B Human Research Ethics Committee, and all subjects provided informed consent (26).

### Pharmacokinetic models of OZ439 and MMV048 in VIS.

Human population pharmacokinetic (PK) modeling of the OZ439 and MMV048 concentration versus time profiles was performed using Monolix (version Monolix 2018R1). A 2-compartment PK model with zero order absorption (see equation S1 in the supplemental material) best described MMV048 concentrations, and a 2-compartment PK model with first-order absorption described the OZ439 concentrations (see equation S2 in the supplemental material). Structural PK model specifications and individual parameter can be found in Supplemental Data Set S1.

### Mathematical models of within-host parasite growth and posttreatment parasite clearance in VIS.

P. falciparum-human infection is described via difference equations able to capture the changing age structure of the parasitemia over time. The difference equations for model S (equations 7 to 9) and model i (equations 10 and 11), with incorporated drug action as specified in Results, are given below (equations 7 to 11). We estimated parasite growth and treatment effects in a two-step sequential process. Firstly, parasite growth parameters were estimated from parasite growth data before treatment. Secondly, we fixed the individual posterior median of parasite growth parameters and individual PK parameters to estimate parameters of drug efficacy (Fig. 1a). Treatment effect *E* incorporates parasite death through treatment as an increase in parasite death rate δ*_p_*. The evaluated treatment models include a direct drug effect model and additional drug-induced growth retardation causing a lengthening of the parasite life cycle length α*_l_*. Model output of model S is the number of circulating parasites *P*_circ_ (equation 14). The parasite multiplication factor pmf can be calculated for model S using the intrinsic parasite multiplication rate *r_p_* and death rate δ*_p_*. It constitutes an estimation of the average number of merozoites that emerge from one infected RBC after a reproduction cycle within an RBC has been finished and successful invasion of new RBCs at parasite age *a *= 1 has completed (equation 7). Details of the parameters are provided in Table 3.

**TABLE 3:** Parameters of the parasite growth and treatment models^*a*^

Parameter	Unit	Description	Bounds [lower, upper]
*i*_pl_	parasites/ml	Parasite density 4 days after inoculation (log transformed)	[1, 15]
μ_ipl_	h	Mean of the initial parasite age distribution	[5, 10]
σ_ipl_	h	SD of the initial parasite age distribution	[2, 20]
δ*_p_*	1/h	Drug-independent parasite death rate	[0.001, 1]
*r_p_*		Parasite replication rate	[40, 80]
α*_l_*	h	Length of the parasite life cycle	Fixed to 39 h
α*_s_*	h	Sequestration age of asexual parasites	Fixed to 25 h
pmf		Parasite multiplication factor	
EC_50_	mg/ml	Drug concn causing 50% of *E*_max_	MMV048, [0.001, 0.8]; OZ439, [1E-6, 0.1]
*E*_max_	1/h	Maximum effect of the drug	[0.0001, 1]
γ		Hill coefficient, steepness of the CE curve	
*k*_ret_	1/h	Growth retardation due to drug treatment	
*k*_ret,max_	1/h	Maximum growth retardation	[0.0001, 1]

a The bounds for parameter estimation were set to include all plausible values based on previously published models (28, 30–32).

### (i) Model S, mechanistic asexual parasite growth model incorporating parasite stages and drug effect.

Our mechanistic model of growth of parasite density *P* over the life cycle of length *a_L_* = 39 h at age *a* and time *t* is given by the following equation:

(7) $$P\left(a,t\right)=\{\begin{array}{c} P\left(a-1,t-1\right)e^{-\delta_{p}},a=2,3,\ldots ,a_{l}\\ r_{p}P\left(a_{l},t-1\right)e^{-\delta_{p}},a=1 \end{array}$$

The direct drug effect on this model is incorporated via *E* (equation 12) representing the drug concentration-dependent increase of parasite death rate δ*_P_*.

(8) $$P\left(a,t\right)=\{\begin{array}{c} P\left(a-1,t-1\right)e^{-\delta_{p}-E},a=2,3,\ldots ,a_{l}\\ r_{p}P\left(a_{l},t-1\right)e^{-\delta_{p}-E},a=1 \end{array}.$$

The direct drug effect model is extended to include drug concentration-dependent growth retardation.

(9) $$P\left(a,t\right)=\{\begin{array}{c} P\left(a,t-1\right)k_{\mathrm{ret}}\frac{\left(1-e^{-\delta_{p}-E-k_{\mathrm{ret}}}\right)}{{\text{δ}}_{p}+E+k_{\mathrm{ret}}}+P\left(a-1,t-1\right)e^{-\delta_{p}-E-k_{\mathrm{ret}}}\text{,}\;a=2,3,\ldots ,a_{l}\\ P\left(a,t-1\right)k_{\mathrm{ret}}\frac{\left(1-e^{-\delta_{p}-E-k_{\mathrm{ret}}}\right)}{{\text{δ}}_{p}+E+k_{\mathrm{ret}}}+r_{p}P\left(a_{l},t-1\right)e^{-\delta_{p}-E-k_{\mathrm{ret}}},a=1 \end{array}$$

### (ii) Model i, exponential parasite growth model incorporating drug effect.

Parasite growth for the exponential model i is modeled on the logarithmic scale with the initial parasite concentration *P*_0_ and parasite growth rate *P_gr_*.

(10) $$\text{ln}(P\left(t\right))=P_{0}+p_{\mathrm{gr}}\;t$$

Treatment is included by decreasing parasite growth rate as follows:

(11) $$\text{ln}(P\left(t\right))=P_{0}+\overset{t}{\underset{0}{\int }}(p_{\mathrm{gr}}-E)$$

### (iii) Additional equations.

In both models, drug effect is given by *E* as follows:

(12) $$E=\frac{E_{\text{max}}C^{\gamma }}{{\text{EC}}_{\text{50}}{}^{\text{γ}}+C^{\gamma }}$$

and growth retardation in mechanistic model S is given by *k*_ret_ as follows:

(13) $$k_{\mathrm{ret}}=\frac{k_{\mathrm{ret}\text{,max}}C^{\gamma }}{{\text{EC}}_{\text{50}}{}^{\text{γ}}+C^{\gamma }}$$

The maximum possible parasite age stage prolongation is therefore given by ((1/1 − *k*_ret,max_ [h^−1^])) − 1[h]) × 60, where 1 h is the original length of the age stage and 60 converts hours into minutes.

In model S, the number of circulating parasites is given by summing parasite concentration up to the parasite age stage α*_s_*, after which parasites sequester.

(14) $$P_{\text{circ}}\left(t\right)=\sum_{a=1}^{a_{s}}P(a,t)$$

The parasite multiplication factor (pmf) over one parasite life cycle with length *a*_l_ for model S is given by

(15) $$\text{pmf}=r_{p}e^{-\delta_{p}a_{l}}$$

The parasite growth rate *p_gr_* of exponential growth model i results in a parasite multiplication factor of

(16) $$\text{pmf}=\text{exp}(p_{\mathrm{gr}}\;a_{l})$$

Under the assumption of exponential parasite growth (model i), the MIC, where parasite replication equals zero, can be calculated with

(17) $$\text{MIC}={\text{EC}}_{\text{50}}\;{\left(\frac{p_{\mathrm{gr}}}{E_{\text{max}}+p_{\mathrm{gr}}}\right)}^{\frac{1}{\gamma }}\;$$

### Parameter estimation.

Parasite growth and pharmacodynamic parameters for humans were estimated in R (version 3.6.0) with package RStan (version 2.18.2) (45) using a Bayesian hierarchical modeling approach. In brief, this means that subject- and/or trial-dependent parameters were defined as a second hierarchy level in addition to the population parameters. Parameters were estimated using a noncentered parameterization approach (46) and then transformed using inverse logit transformation within prespecified lower and upper bounds (47) based on biological background information (Table 3). Prior distributions for the population mean parameters were given by standard normal distributions before the logit transformation. Priors for the interindividual variability were defined by the Cholesky factors of the correlation matrices using a Cholesky LKJ correlation distribution with a shape parameter of 2 for efficiency and computational stability (47). We ran five chains with 4,000 iterations each of which 2,000 were used as a burn-in. The posterior samples were cumulated over all chains to illustrate the joint and marginal posterior distributions in Fig. S7 to S12 in the supplemental material. The 95% credible interval of each parameter is given by the 2.5% and 97.5% quantiles of the posterior distribution (see Fig. S3 to S6 in the supplemental material). The M3 method was used for dealing with parasite measurements below the lower limit of quantification of 111 parasites/ml.

Models were evaluated using the R package bayesplot (version 1.6.0) and loo (version 2.1.0), and model selection was based on the Watanabe-Akaike information criterion (WAIC) (48) and the effective sample size *n*_eff_, an estimate of the number of independent draws from the posterior distribution. *n*_eff_ was required to be over 0.1 for all parameters. Additionally, divergences in any of the chains were evaluated visually, and the convergence criteria $\hat{R}\;$ was calculated (potential scale reduction statistic should be less than 1.05). Posterior predictive checks were performed to visually assess how well the model fits the parasitological data.

### Model simulation and analysis: parasite clearance.

We used the well-calibrated models to estimate efficacy index parasite clearance rate and log_10_ PRR_48_ for a range of drug doses and regimes in both murine and human testing systems (Fig. 1b, parasite clearance analysis).

Simulation and subsequent analysis of murine P. berghei-NMRI and P. falciparum-SCID infection were executed in a pooled manner: one set of parameters was drawn and simulated over all doses for each experiment. Population parameter distributions are defined as a log-normal distribution LN(μ, 0.2μ) with the previously estimated mean μ (see Table S5 in the supplemental material for a summary) (15). The experimental parameters infectivity parameter β and initial percentage of human RBCs *H*_0_ were drawn from the pool of estimated parameter values. Simulations were performed using R package IQRtools (version 0.9.999).

P. falciparum-human infection was simulated with parameter variability implemented on a trial and subject level. Within one trial, subjects were allocated the subject-specific parameters *i*_pl_ and δ*_p_* and shared the parasite related parameters μ_ipl_, σ_ipl_, and *r_p_*. Parameters were drawn from the estimated variance-covariance within the bounds specified for estimation (Table 3). Simulations were performed in R. Human PK parameters were fixed to population-level parameters. Additional information on the simulations can be found in Table S4 in the supplemental material. Parameters not previously estimated were fixed to values previously reported (15, 26). Simulation results were processed to extract the parasite clearance rate as described previously (49). Potential lag phases after treatment at the beginning of the clearance curve and tail phases at the end of the clearance curve are excluded from analysis as described previously (49). The clearance rate is extracted from the clearance curve on the log scale using linear regression and corresponds to the slope of the parasite clearance curve.

### Model simulation and analysis: sensitivity of parasite clearance to host-parasite and drug dynamics.

A global sensitivity analysis was performed to assess the sensitivity of parasite clearance after treatment to the parameters describing drug action and parasite growth. We performed a global sensitivity analysis by decomposition of the variance of model output (in this case, parasite clearance rate) via Sobol analysis (50). Calculation of the first-order indices and total effect indices for all model parameters allows assessment of individual and combined parameter contributions to the variance of parasite clearance rate across the whole parameter space (51). As Sobol analysis is computationally intensive due to the required number of points across the input parameter space (*n* = 200,000) and bootstrap replicates (*n* = 1,000), we reduced computational time needed to simulate the parasite-PKPD models by training emulators of the original models. We thus trained a Gaussian process model on simulation output for each of the parasite growth models and doses analyzed using R package hetGP (version 1.1.2). We normalized input parameters and output to be between 0 and 1 due to the large differences in scales. The criterion for acceptance of our trained model was out of sample prediction with a predictive accuracy of *R*^2^ > 0.97. The sensitivity analysis was performed using the function soboljansen in the R package sensitivity (version 1.16.2) on the trained Gaussian-process model. Parameters contributing under 1% were excluded from further analysis. Remaining parameters were summarized into parameters of host, parasite, host-parasite, and drug dynamics for the visualization of results according to Table 2.

### Data availability.

The data sets analyzed during the current study are available from the corresponding author on request and with permission of Medicines for Malaria Venture.

## Supplementary Material

Supplemental file 1

Supplemental file 2

Supplemental file 3
