# Supplementary material for: Parasite-Host Dynamics throughout Antimalarial Drug Development Stages Complicate the Translation of Parasite Clearance
Source: Antimicrob Agents Chemother. 2021 Mar 18;65(4):e01539-20. doi: 10.1128/AAC.01539-20 (PMC8097426; doi:10.1128/AAC.01539-20)
Supplement: Supplemental file 1 [file AAC.01539-20-s0001.pdf]

Parasite-host dynamics throughout antimalarial drug development stages complicate the translation of parasite clearance

Running title: Parasite clearance in antimalarial drug development

AUTHORS: Lydia Burgert[1, 2], Sophie Zaloumis[3], Saber Dini[3], Louise Marquart [4], Pengxing Cao [5], Mohammed Cherkaoui[6], Nathalie Gobeau[6], James McCarthy[4], Julie A. Simpson[3], Jörg J. Möhrle[1, 2, 6], Melissa A. Penny*[1, 2]
AFFILIATIONS: [1] Swiss Tropical and Public Health Institute, Basel, Switzerland, [2] University of Basel, Basel, Switzerland, [3] Centre for Epidemiology and Biostatistics, Melbourne School of Population and Global Health, University of Melbourne, Melbourne, Australia, [4] QIMR Berghofer Medical Research Institute, Brisbane, Australia, [5] School of Mathematics and Statistics, University of Melbourne, Melbourne, Australia,[6] Medicines for Malaria Venture, Geneva, Switzerland

*Corresponding author: melissa.penny@unibas.ch

Email of other authors:
lydia.burgert@swisstph.ch
sophiez@unimelb.edu.au
saber.dini@unimelb.edu.au
Louise.Marquart@qimrberghofer.edu.au
pengxing.cao@unimelb.edu.au
cherkaouim@mmv.org
gobeaun@mmv.org
J.mccarthy@uq.edu.au
julieas@unimelb.edu.au
moehrlej@mmv.org

1. Data for drug efficacy in VIS

Table S 1: Details of parasite clearance data used for estimating parameters of drug efficacy. The cohort name corresponds to the names provided in a previous analysis of parasite growth data from malaria Volunteer Infection Studies (VIS)[1]. The cohort number is linked to the parasite growth parameter estimation and corresponds to the cohorts shown in Fig. S7 and Fig. S8. The parasite growth (PG) and pharmacodynamic (PD) IDs are identifiers used in estimating the respective parameters.

Drug	Clinical trial registry	Cohort name*	No. Subj.	Dose [mg]	No. Cohort	PG ID	PD ID
MMV048	NCT02281344	MMV048_PIB: CH1	6	20	15	96-101	1-6
	NCT02783833	MMV049_PartB: CH1	7	40	26	163-170	7-13
	NCT02783833	MMV049_PartB: CH2	7	80	27	170-177	14-20
OZ439	ACTRN12612-000814875	OZ439:CH1	8	100	4	23-30	1-7
	ACTRN12612-000814875	OZ439:CH2	8	200	5	31-38	8-16
	ACTRN12612-000814875	OZ439:CH3	8	500	6	39-46	17-24

2. Pharmacokinetic (PK) models

Model structures

The drug-concentration after dosing with dose D [mg] is given by a two-compartment PK model with zero-order absorption for MMV048 and a two-compartment model with first-order absorption for OZ439. Below we provide the analytical solutions for both models (detailed by Monolix, http://mlxtran.lixoft.com/libraries). Individual PK parameters were used for estimation of drug action parameters in each human volunteer. Subsequent simulation utilised the population parameters. Both individual parameter and population level parameters can be found in Supplementary File 1. The PK parameter models and estimates were kindly provided by MMV, Geneva.

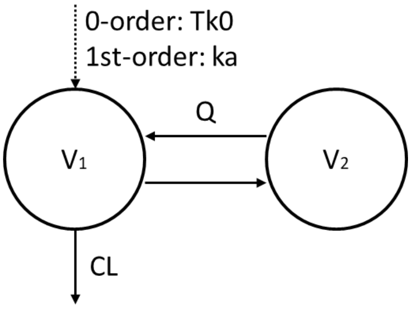

Figure S1: General schematic representation of a 2-compartment model with 0- or 1st order absorption process. V1 is the central compartment and V2 the peripheral compartment, rates of flow into and out of compartments are calculated with the equations given below.

<u>2 compartment model with zero-order absorption</u>

$$C(t) = \begin{cases} \frac{D}{T_{k0}}\left[\frac{A}{\alpha}(1-e^{-\alpha t}) + \frac{B}{\beta}\left(1-e^{-\beta t}\right)\right] \text{ if } t \leq T_{k0}, \text{ else} \\ \frac{D}{T_{k0}}\left[\frac{A}{\alpha}\left(1-e^{-\alpha T_{k0}}\right)e^{-\alpha(t-T_{k0})} + \frac{B}{\beta}\left(1-e^{-\beta T_{k0}}\right)e^{-\beta(t-T_{k0})}\right] \end{cases} \tag{S1}$$

$$k_{10} = \frac{CL}{V_1};\ k_{12} = \frac{Q}{V_1};\quad k_{21} = \frac{Q}{V_2}$$

$$\beta = 0.5(k_{10} + k_{12} + k_{21} - \sqrt{(k_{10} + k_{12} + k_{21})^2 - 4k_{21}k_{10}};\quad \alpha = \frac{k_{21}\,k_{10}}{\beta};$$

$$A = \frac{1}{V_1}\frac{\alpha - k_{21}}{a-\beta};\quad B = \frac{1}{V_1}\frac{\beta - k_{21}}{\beta - a}$$

<u>2 compartment model with linear absorption and lag-time</u>

$$C(t) = \begin{cases} 0 \text{ if } t \leq T_{lag}, \text{ else} \\ D\left[Ae^{-\alpha\,(t-T_{lag})} + Be^{-\beta\,(t-T_{lag})} - (A+B)e^{-k_a\,(t-T_{lag})}\right] \end{cases} \tag{S2}$$

$$k_{10} = \frac{CL}{V_1};\ k_{12} = \frac{Q}{V_1};\quad k_{21} = \frac{Q}{V_2}$$

$$\beta = 0.5(k_{10} + k_{12} + k_{21} - \sqrt{(k_{10} + k_{12} + k_{21})^2 - 4k_{21}k_{10}};\quad \alpha = \frac{k_{21}\,k_{10}}{\beta};$$

$$A = \frac{k_a}{V_1}\frac{k_{21} - \alpha}{(k_a - a)(\beta - a)};\quad B = \frac{k_a}{V_1}\frac{k_{21} - \beta}{(k_a - \beta)(a - \beta)}$$

3. Parameter estimation

Table S2: Selection of the parasite growth model. The mechanistic parasite growth models S1-S3 differ in their hierarchical assignment of parameters to cohort and subject specific parameters. Based on the widely applicable information criterion (WAIC) we selected *model S3* for further analysis of drug action (referred to as *model S*). The correlation between $\boldsymbol{r_p}$ and $\boldsymbol{\delta}_{\mathrm{p}}$ was calculated based of draws from the population posterior prediction.

**Model**	**Hierarchy**		**WAIC (SE)**	**Correlation between $\boldsymbol{r_p}$ and $\boldsymbol{\delta}_{\mathrm{p}}$**
	**Cohort**	**Subject**		
S1	-	ipl, δ, r_p, μ_{ipl} and σ_{ipl}	2024 (41)	0.75 (p≤0.01)
S2	r_p	ipl, μ_{ipl}, σ_{ipl} and δ	1850 (39)	0.84 (p≤0.01)
S3	r_p, μ_{ipl} and σ_{ipl}	ipl and δ	**1679 (40)**	0.84 (p≤0.01)
i		ipl and r_p	2574 (24)	-

Table S3: Selection of the drug effect model for treatment with MMV048 and OZ439. Per drug action model, four different hill constants were tested and models were selected per parasite growth (PG) model based on WAIC and additional observations made during the fitting process.

**Drug**	**PG Model**	**Drug action**	**WAIC (SE)**	**hill**	**Additional observations after model fitting**
**MMV048**	*S3*	Direct effect	1031 (41)	1	Directly low dose treatment model oscillation is out of phase with data
			992 (36)	2	
			955 (33)	5	
			**952 (34)**	**7**	
	S3	Direct effect+ growth retardation	901 (46)	1	Parasite oscillation after treatment captured in model
			835 (36)	2	
			**774 (25)**	**5**	
			779 (25)	7	
	i	Direct effect	1055 (30)	1	Lower doses not well captured
			1012 (25)	2	
			973 (19)	5	
			**969 (19)**	**7**	
**OZ439**	*S3*	Direct effect	866 (26)	1	Treatment effect after lower doses not well captured.
			850 (26)	2	
			**841 (27)**	**5**	
			842 (27)	7	
	S3	Direct effect+ growth retardation	685 (27)	1	Able to capture treatment effect after lower doses
			**657 (28)**	**2**	
			657 (31)	5	
			659 (31)	7	
	i	Direct effect	828 (18)	1	No drug effect for low doses
			813 (18)	2	
			**807 (19)**	**5**	
			809 (19)	7	

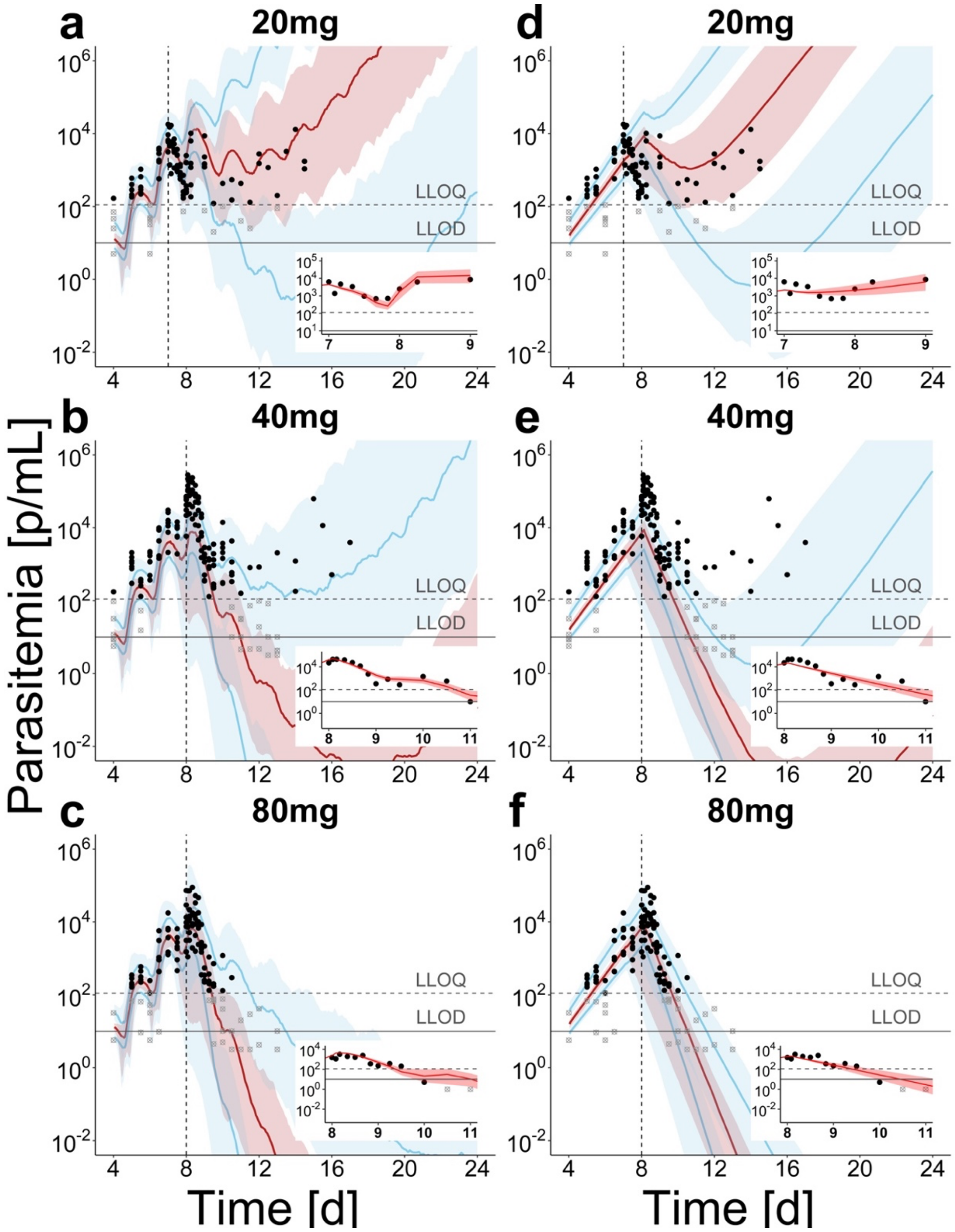

Figure S2: Population prediction after treatment with MMV048 in *P. falciparum*-human infection for mechanistic growth *model S* (a-c) and exponential *model i* (d-f) The median (red) and 90th percentile (blue) with credible intervals over 100 trials with 20 subjects is compared to individual parasite densities (•) in the respective treatment group. For each treatment group, parasite clearance of a typical subject (Subject 5, 13, and 17) immediately after treatment is illustrated with individual prediction (for all subjects see Supplementary Figure S3 and S4). The lower doses of 20mg and 40 mg MMV048 only show transient drug effect with parasitemia recrudescing in all subjects (see individual data in Supplementary Figure S3 and S4).In the 20 mg dose group **(a, d)**, *model S* predicts later recrudescence. After treatment with 40 mg **(b, e)**, treatment effects are overestimated for *model i*. Both models predict no recrudescence during the 24 days of follow-up for the highest dose (80mg **(c, f)**). However, predicted parasite clearance shows a larger range with *model S*. Vertical line (---) indicates time point of treatment and the horizontal lines LLOQ of 111 p/mL and LLOD of 10 p/mL.

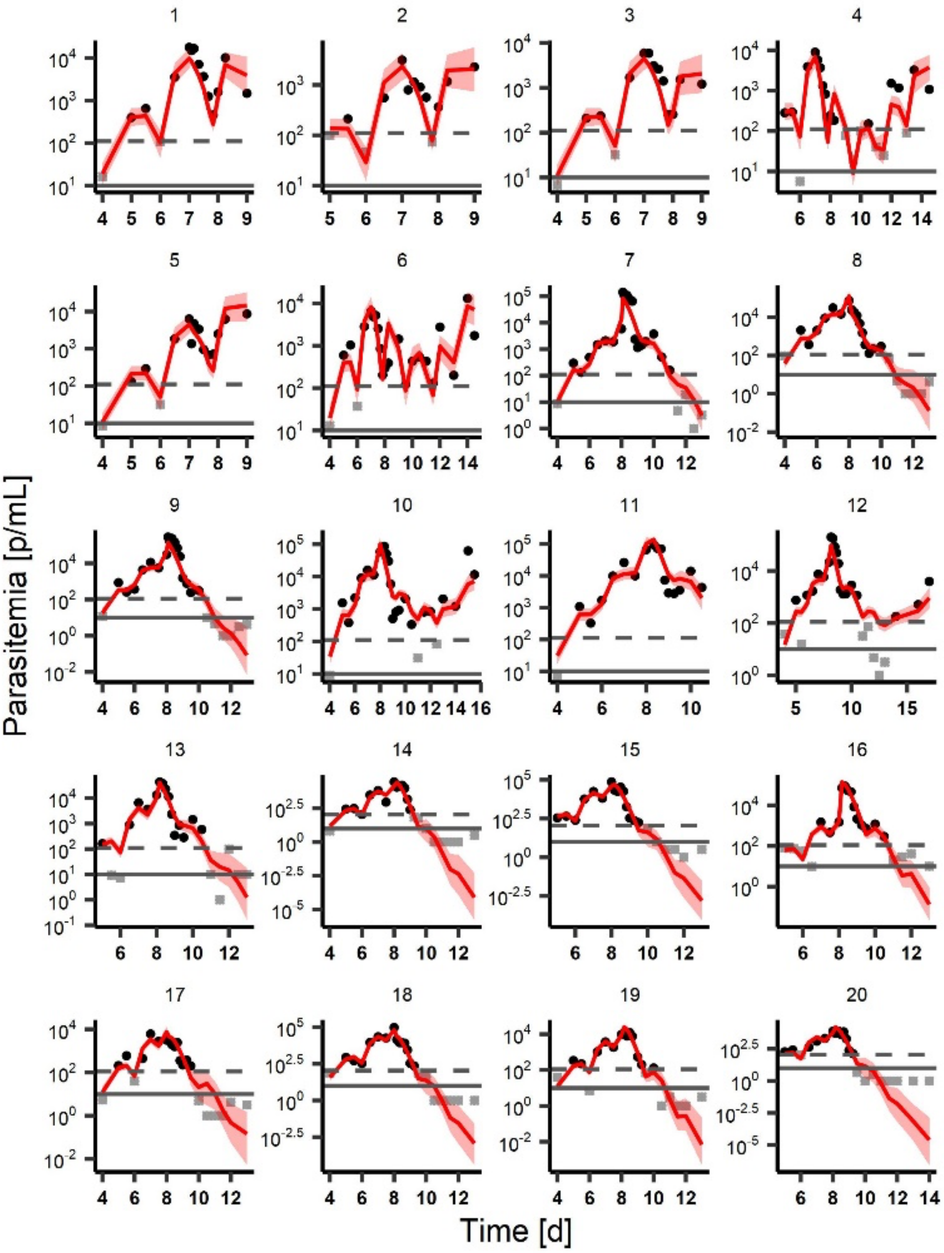

Figure S3: Individual posterior predictive check of parasite growth *model S* with a direct drug effect and additional growth retardation for the 20 subjects treated with MMV048 in VIS. Subjects are indicated by numbered title of each panel and were treated with 20 mg (Subjects 1-6, Cohort 15, day 7), 40 mg (Subjects 7-13, Cohort 26, day 8), and 80 mg (Subj. 14-20, Cohort 27, day 8) respectively. The periodicity in parasitemia is captured for all subjects before treatment. The model is able to describe treatment effects including recrudescence over all dose ranges. The median predicted parasitemia and its 90% credible interval are illustrated in red, with data above the LLOQ (---) in black and below in grey. Posterior predictions were generated as specified in Material and Methods.

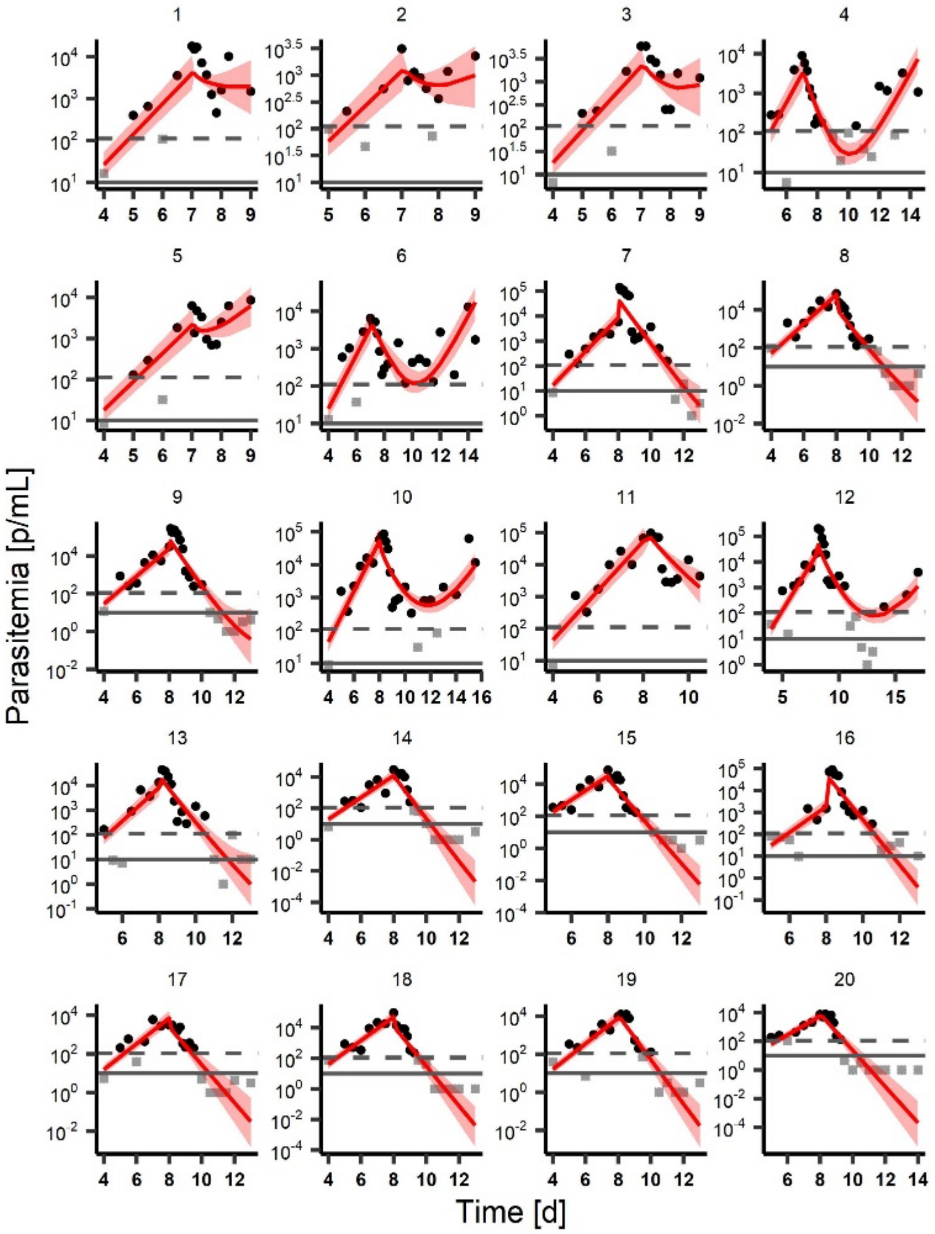

Figure S4: Individual posterior predictive check of parasite growth *model i* with a direct drug effect for the 20 subjects treated with MMV048 in VIS. Subjects are indicated by numbered title of each panel and were treated with 20 mg (Subjects 1-6, Cohort 15, day 7), 40 mg (Subjects 7-13, Cohort 26, day 8), and 80 mg (Subjects 14-20, Cohort 27, day 8) respectively. General trends in treatment are captured well for the two higher doses. After treatment with 20 mg, predictive intervals for subj. 1-5 are wide, capturing scenarios of increase and decrease of parasitemia after treatment. The median predicted parasitemia and its 90% credible interval are illustrated in red, with data above the LLOQ (---) in black and below in grey. Posterior predictions were generated as specified in Material and Methods.

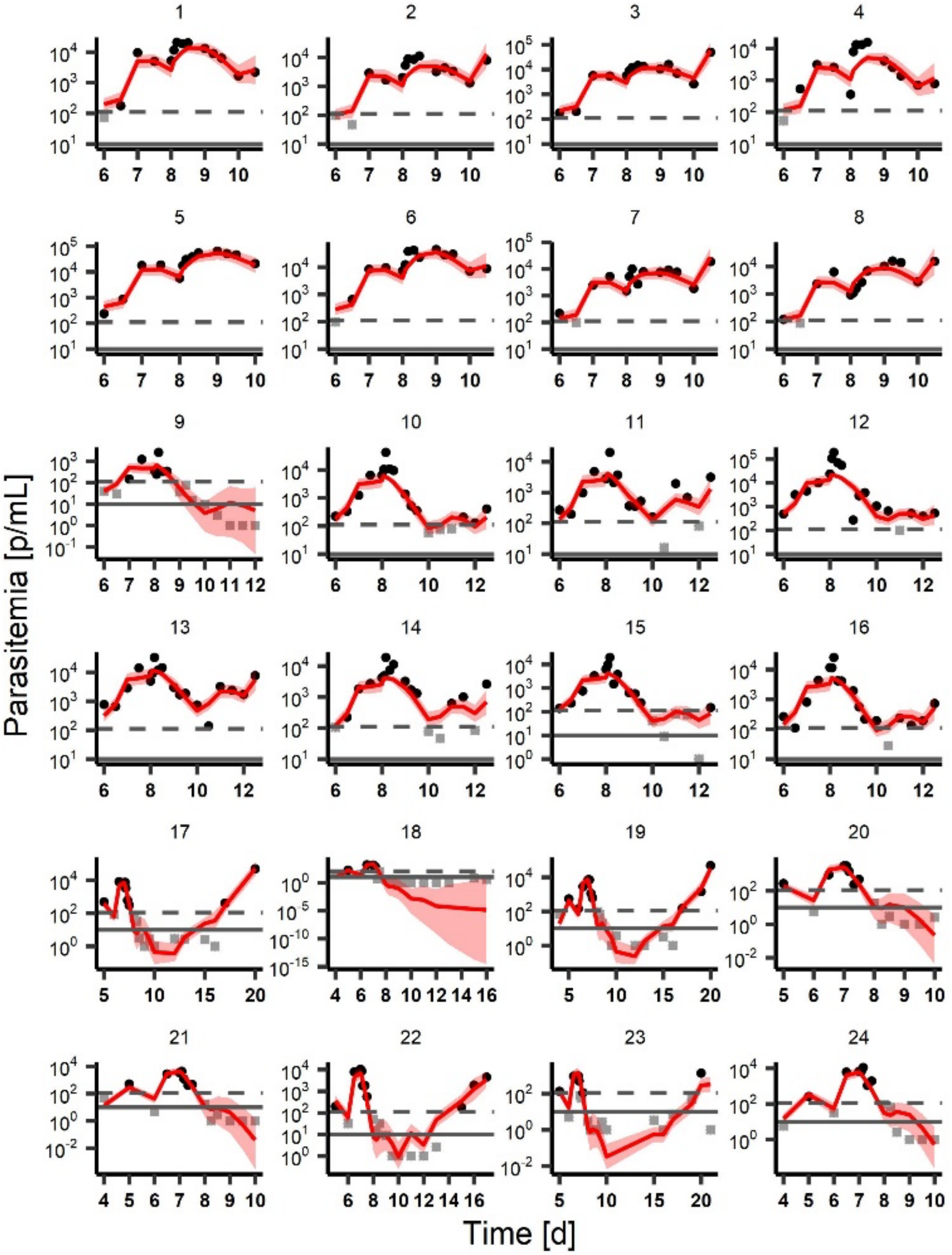

Figure S5: Individual posterior predictive check of parasite growth *model S* with a direct drug effect and additional growth retardation for the 24 subjects treated with OZ439 in VIS. Subjects are indicated by numbered title of each panel and were treated with 100 mg (Subjects 1-8, Cohort 4, day 8), 200 mg (Subjects. 9-16, Cohort 5, day 8), and 500 mg (Subjects 17-24, Cohort 6, day 7) respectively. After treatment with 100 mg, the model is able to capture the transient treatment effect of an initial increase directly after treatment and slight decrease around day 9. The model captures individual occurrence of recrudescence events well (Subj. 17, 19, 22, 23). The median predicted parasitemia and its 90% credible interval are illustrated in red, with data above the LLOQ (---) in black and below in grey. Posterior predictions were generated as specified in Material and Methods.

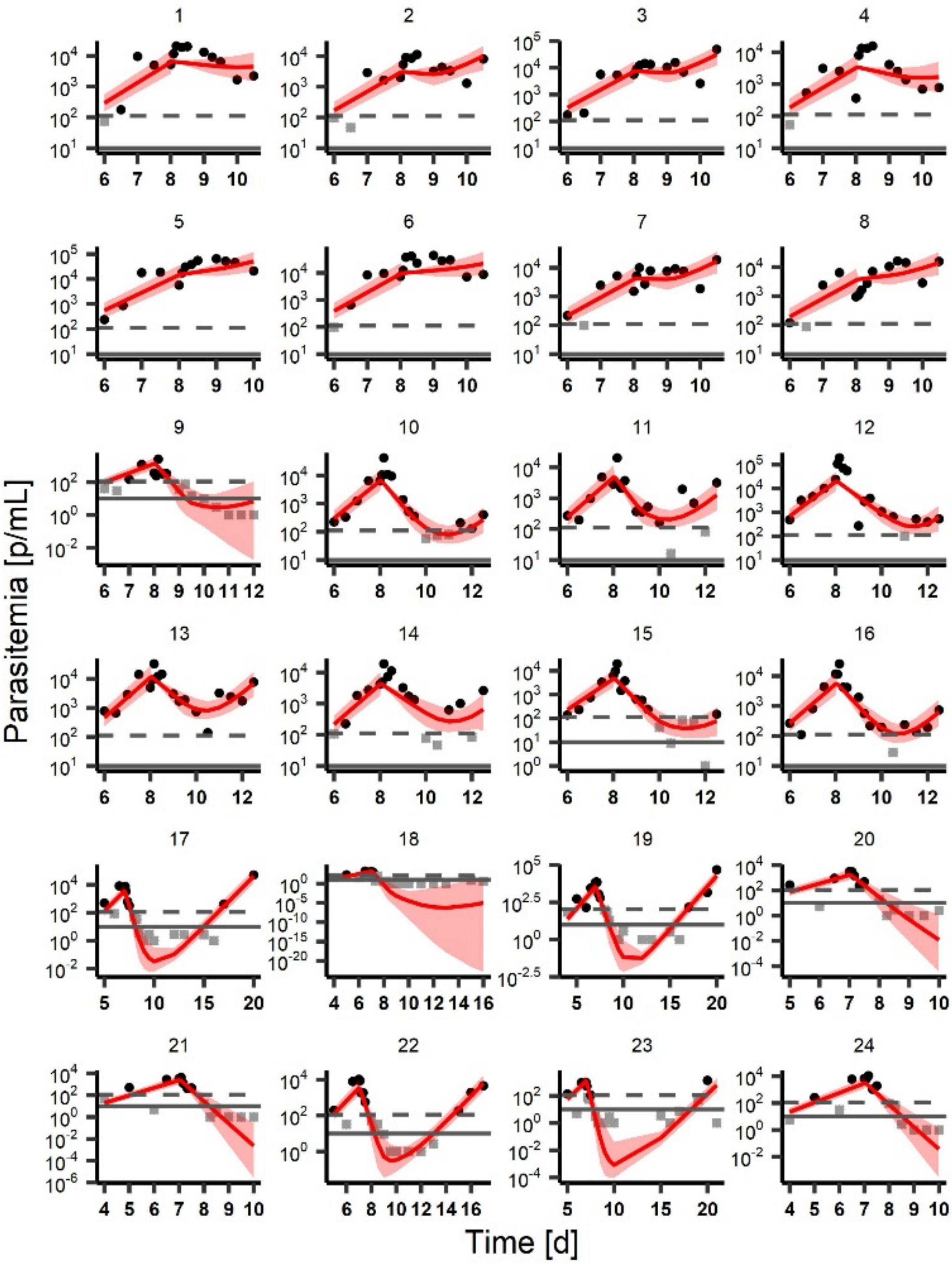

Figure S6: Individual posterior predictive check of parasite growth *model i* with a direct drug effect for the 24 subjects treated with OZ439 in VIS. Subjects are indicated by numbered title of each panel and were treated with 100 mg (Subjects 1-8, Cohort 4, day 8), 200 mg (Subjects 9-16, Cohort 5, day 8), and 500 mg (Subjects 17-24, Cohort 6, day 7) respectively. Model predictions are not capturing the transient treatment effect after treatment with 100 mg. The median predicted parasitemia and its 90% credible interval are illustrated in red, with data above the LLOQ (---) in black and below in grey. Posterior predictions were generated as specified in Material and Methods.

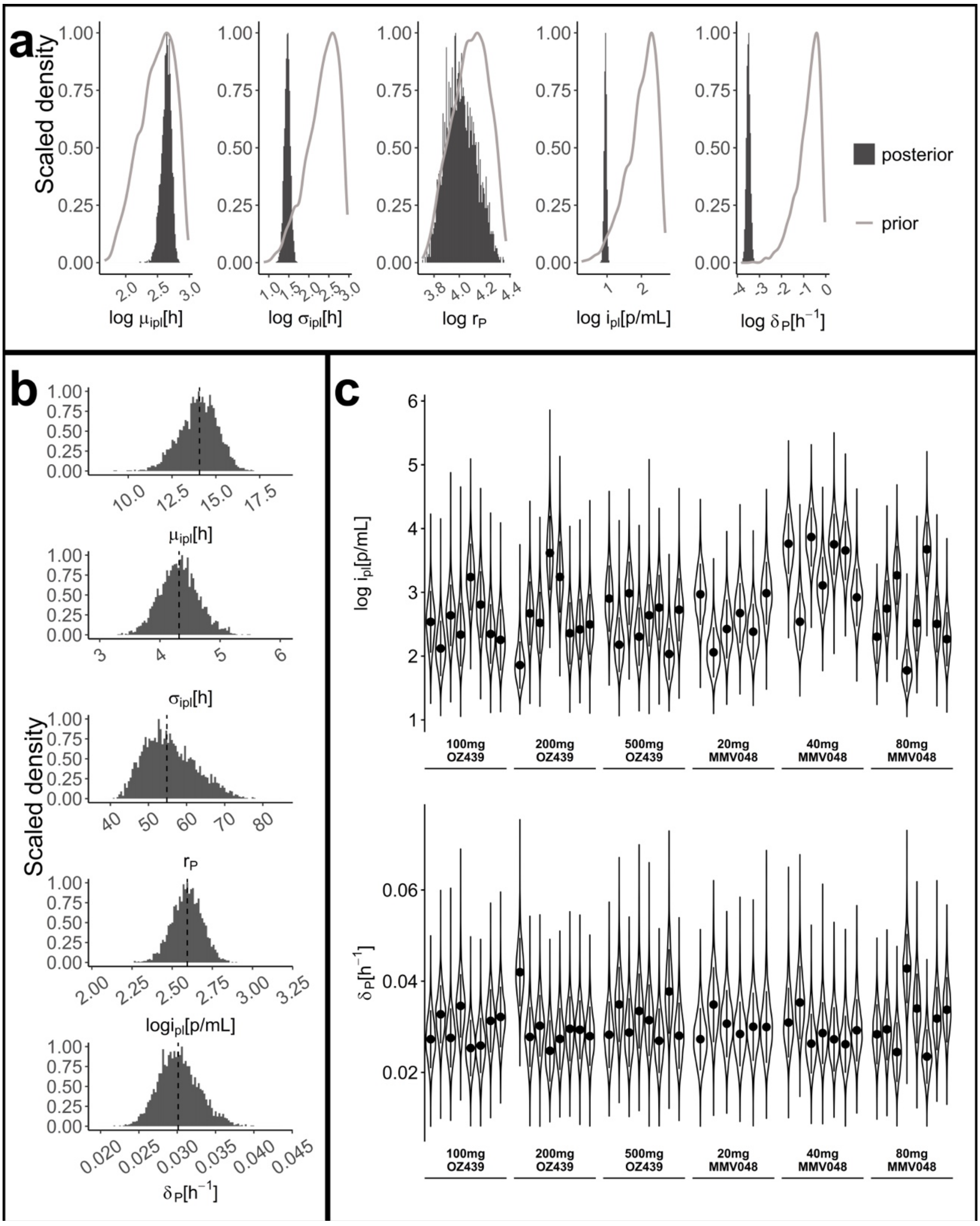

Figure S7: Prior and marginal posterior parameter distributions for mechanistic parasite growth *model S3* in *P. falciparum*-human infection over all cohorts and the subjects of interest. (a) Comparison of the scaled density of prior and posterior distribution, (b) population parameter estimates with their median, and (c) posterior distributions on a cohort or level (upper three panels) or for the subjects in the MMV048 and OZ439 cohorts. The parasite replication rate r_p exhibits week identifiability with congruent prior and posterior distributions (a) and posterior distributions of individual cohorts spanning the whole range between upper and lower bounds.

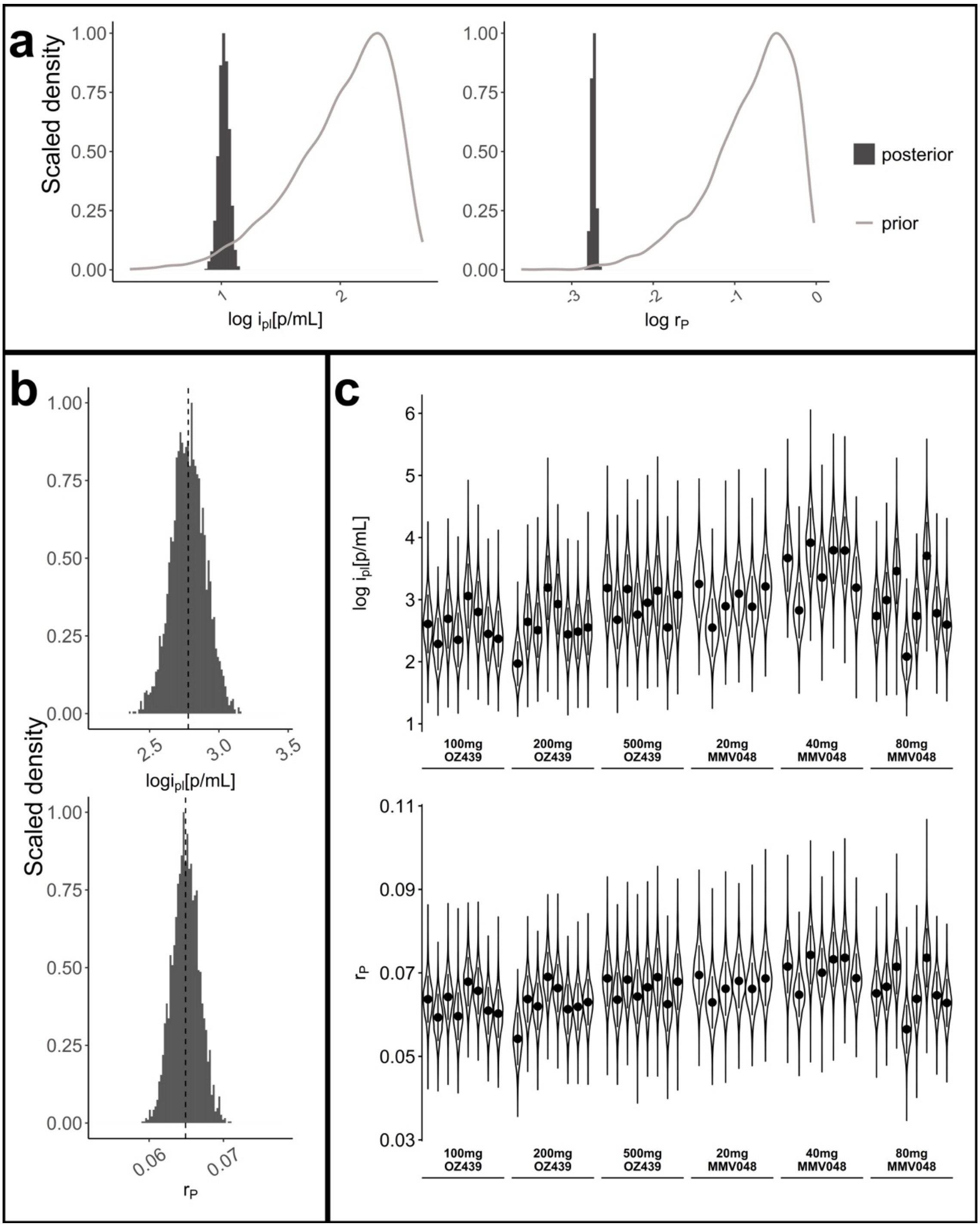

Figure S8: Prior and marginal posterior parameter distributions for exponential parasite growth *model i* in *P. falciparum*-human infection over all cohorts and the subjects of interest. (a) Comparison of the scaled density of prior and posterior distribution, (b) population parameter estimates with their median, and (c) posterior distributions on a cohort or level (upper three panels) or for the subjects in the MMV048 and OZ439 cohorts.

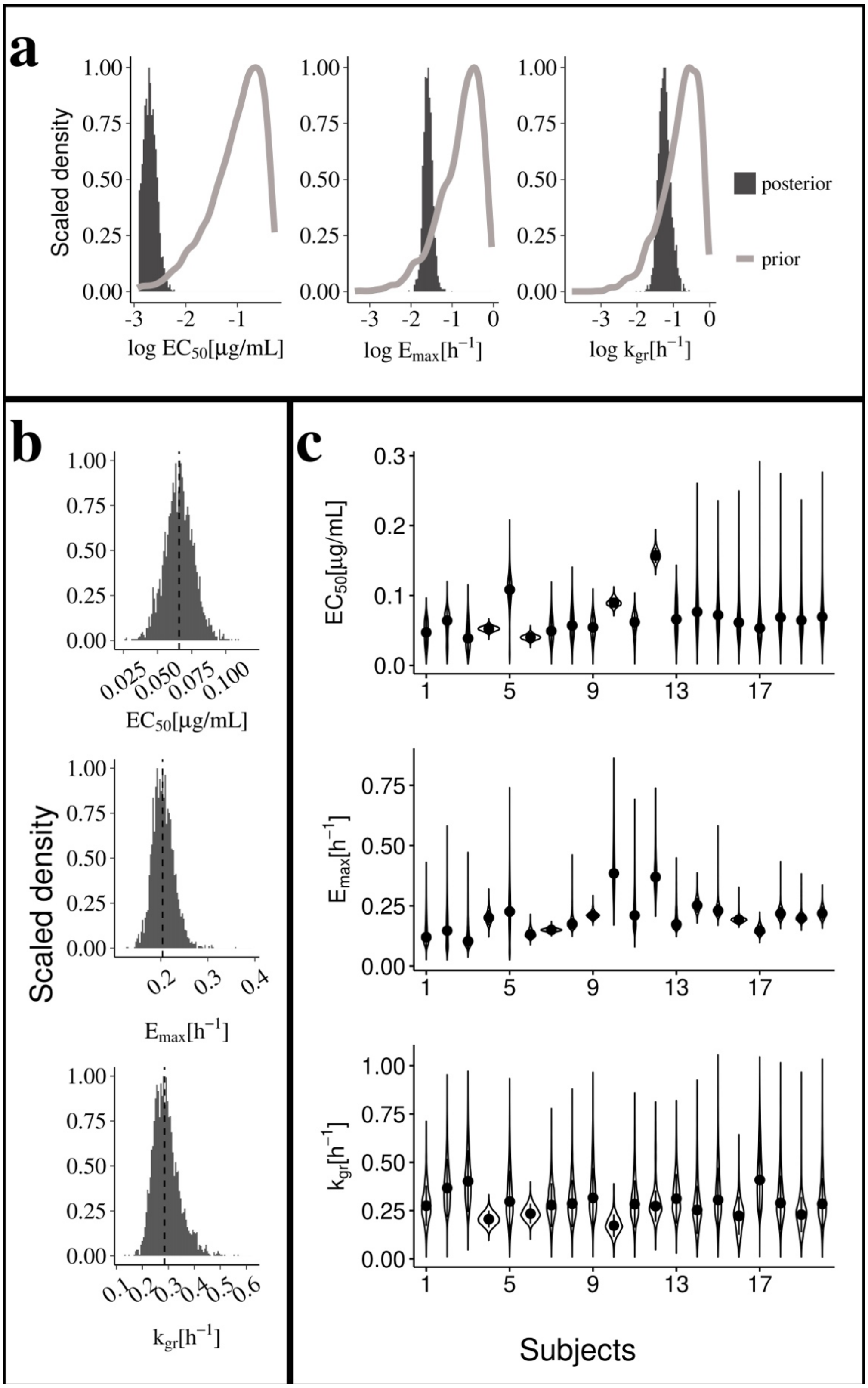

Figure S9: Prior and marginal posterior parameter distributions for drug efficacy parameters after treatment with MMV048 in *P. falciparum*-human infection for mechanistic growth *model S*-growth retardation (a) Comparison of the scaled density of prior and posterior distribution, (b) population parameter estimates with their median, and (c) posterior distributions on an individual level. There are identifiability issues of EC_{50} for subjects 14-17. As not all subjects in this dosing group (80mg) exhibited recrudescent, information on the efficacy of sub-curative drug concentration is missing for this dose group.

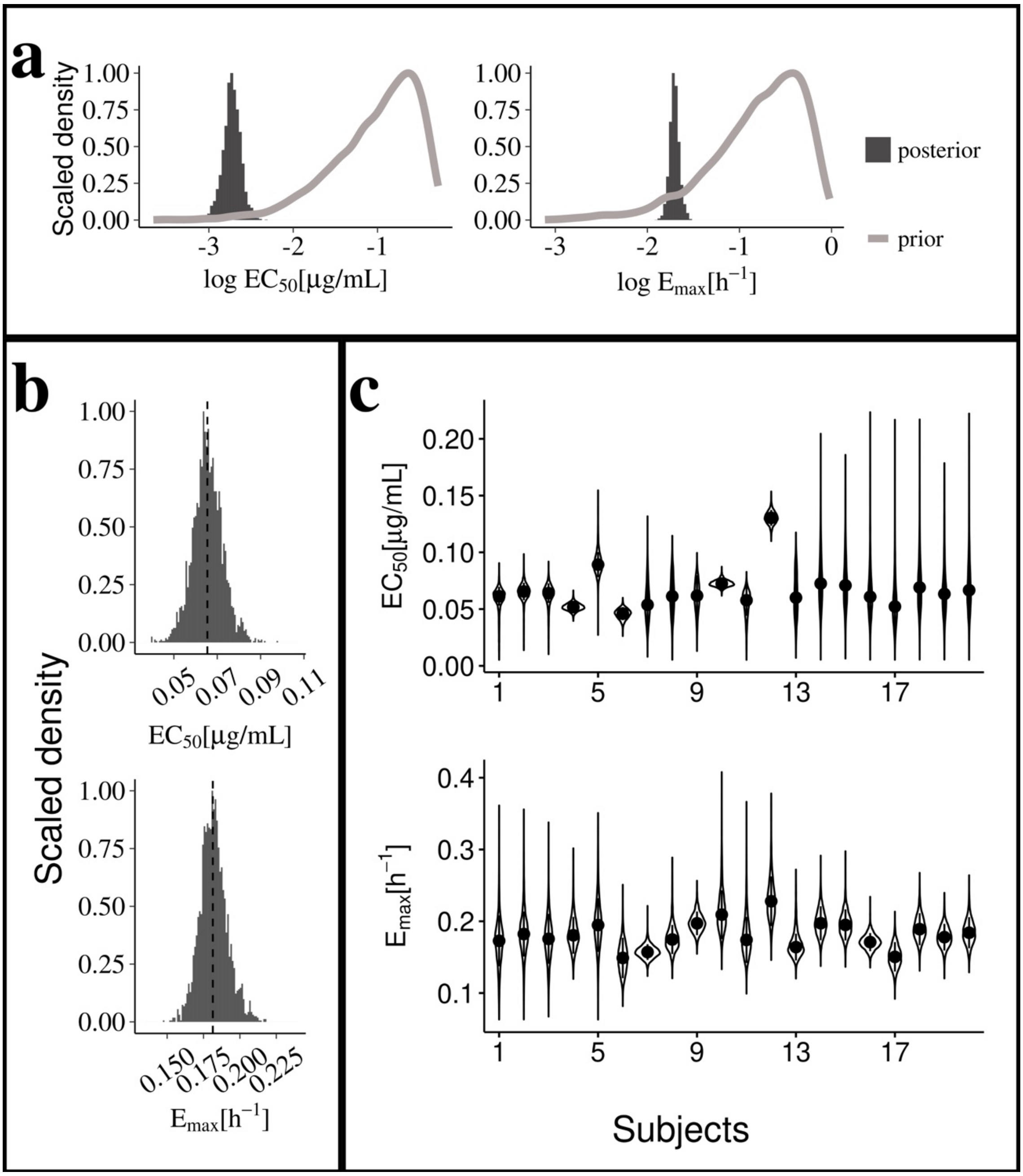

Figure S10: Prior and posterior marginal parameter distributions for drug efficacy parameters after treatment with MMV048 in *P. falciparum*-human infection for exponential growth *model i*-direct effect. There are identifiability issues of EC_{50} for subjects 14-17. As not all subjects in this dosing group (80mg) exhibited recrudescent, information on the efficacy of sub-curative drug concentration is missing for this dose group.

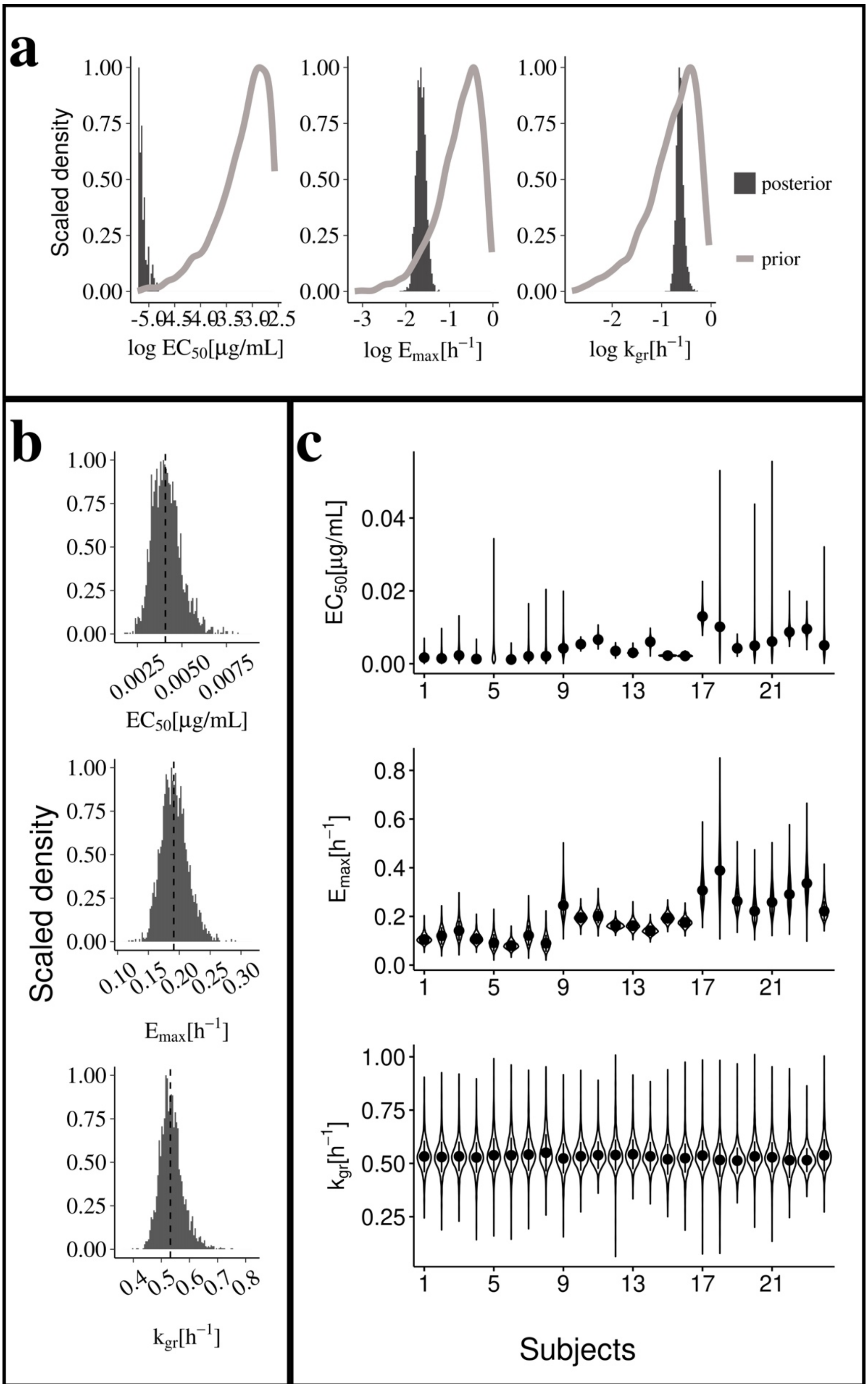

Figure S11: Prior and marginal posterior parameter distributions for drug efficacy parameters after treatment with OZ439 in *P. falciparum*-human infection for mechanistic growth *model S* with drug induced growth retardation. (a) Comparison of the scaled density of prior and posterior distribution, (b) population parameter estimates with their median, and (c) posterior distributions on an individual level.

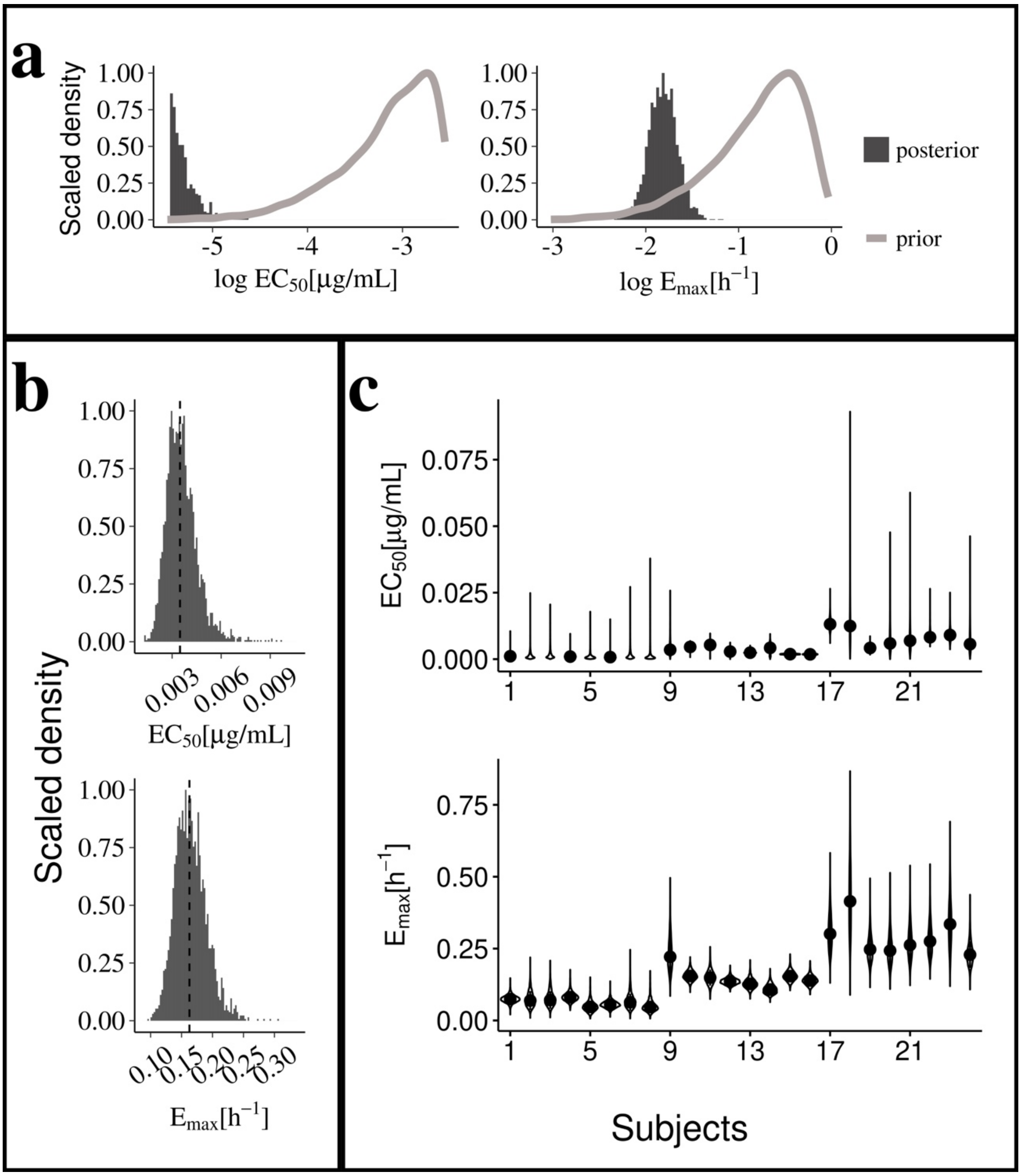

Figure S12 Prior and marginal posterior parameter distributions for drug efficacy parameters after treatment with OZ439 in *P. falciparum*-human infection for exponential growth *model i* and direct drug effect. (a) Comparison of the scaled density of prior and posterior distribution, (b) population parameter estimates with their median, and (c) posterior distributions on an individual level.

Table S4: Specification of simulation set-up for murine and human infection. Inputs into the model including dosing, parasite inoculum, and injections of human erythrocytes (hRBCs) were carried out as specified below.

	***P. berghei* - NMRI and *P.falciparum* -SCID**	***P. falciparum* - human**
Level of variability	Trial	Trial and subject
No. trials	1000	100
No. subjects/ dose/ trial	1	20
Simulation length [d]	20	30
No. doses	1	1
Dosing Time [d]	4	8
Parasite inoculum	*P. berghei*: 2e7 parasites<br>*P. falciparum*: 3e7 parasites	Sampled i_{pl} [p/mL]
Body weight	0.021 kg	-
Blood volume	0.0025 L	-
Additional specification	P. falciparum: injection of 4.55e9 hRBC every 24 hours for the first week, then every 48 hours	-
Parameter sampling	Parameters previously estimated (1) were sampled from LN(μ,0.2μ) (Table S5)	Estimated variance-covariance matrix within parameter bounds [a, b] (Table 1)

Table S5: Summary of parameter estimates for murine malaria experiments of *P. berghei* - NMRI infection (*model a-model e*) and *P. falciparum*- SCID infection (*model f-model i*)(1). PD model selection, namely the best fitting structural drug action model for each drug are indicated in the drug action parameter column, with Cl_Y for delayed parasite clearance model and k_R for a turnover model. The parameters listed were sampled from a log-normal distribution LN(μ,0.2μ) for simulations. All other parameters were fixed to values specified in the Supplementary Material of previous growth analysis for murine malaria infection (1). * Range of values for infectivity parameter β and the initial percentage of human RBCs H_0 estimated over all experiments.

	Parasite growth parameter		Drug action parameter		
**Model**	**Parameter [Unit]**	**Mean estimate μ**	**Parameter**	**MMV-048**	**OZ439**
**Model a-base**	β * [cells/mLh]	6.7e-11 - 1.7e-10	EC_{50} [ng/mL]	3.7e2	49
	r	14.5	E_{max} [1/h]	0.61	0.93
	ω	0.99	Cl_Y or k_R [1/h]	Cl_Y=0.039	k_R=0.013
**Model b-bystander**	β* [c/mLh]	1.9e-10 - 5e-10	EC_{50} [ng/mL]	2.8e2	49
	r	11.5	E_{max} [1/h]	0.39	0.94
	ω	0.28	Cl_Y or k_R [1/h]	Cl_Y=0.036	k_R=0.013
	y_{max} [1/h]	0.02			
	ky_{50} [10^{10}c/mL]	7.7e-5			
**Model c-comp. erythr.**	β* [c/mLh]	7.1e-11-1.7e-10	EC_{50} [ng/mL]	3.7e2	42
	r	14.5	E_{max} [1/h]	0.61	0.28
	ω	0.99	Cl_Y or k_R [1/h]	Cl_Y=0.039	k_R =0.060
**Model d-impaired maturation**	β* [c/mLh]	9.8e-11 - 2.4e-10	EC_{50} [ng/mL]	3.1e2	46
	r	13.0	E_{max} [1/h]	0.45	0.84
	ω	0.74	Cl_Y or k_R [1/h]	Cl_Y=0.041	k_R=0.015
	$k_{l,50}$[10^{10}c/mL]	0.12			
**Model e-reticulocytes**	β *[c/mLh]	7.1e-11 - 1.2e-10	EC_{50} [ng/mL]	2.2e2	46
	r	10.6	E_{max} [1/h]	0.19	0.91
	ω	0.78	Cl_Y or k_R [1/h]	k_R=0.055	k_R=0.015
	ε	5.1			
**Model f - const. RBC decay**	β*[c/mLh]	2.4e-10 - 1.6e-9	EC_{50} [ng/mL]	1.2e2	75
	H_0*	0.40 – 0.69	E_{max} [1/h]	0.090	0.26
	r	21.3	Cl_Y or k_R [1/h]	Cl_Y=0.071	k_R=0.016
	λ[1/h]	0.010			
	γ_{max} [1/h]	0.44			
	$k\gamma_{50}$ [c/mL]	0.24			
	ω	0.29			
	φ [1/h]	0.030			

Table S5: Summary of parameter estimates for murine malaria experiments(1) of *P. berghei* -NMRI infection (*model a-model e*) and *P. falciparum*- SCID infection (*model f-model i*). Continued

Model	Parasite growth parameter		Drug action parameter		
	**Parameter [Unit]**	**Mean estimate μ**	**Parameter**	**MMV-048**	**OZ439**
**Model f - const. RBC decay**	β^*[c/mLh]	2.4e-10 - 1.6e-9	EC_{50} [ng/mL]	1.2e2	75
	H_0*	0.40 – 0.69	E_{max} [1/h]	0.090	0.26
	r	21.3	x[1/h]	Cl_Y=0.071	k_R=0.016
	λ[1/h]	0.010			
	γ_{max} [1/h]	0.44			
	$k\gamma_{50}$ [c/mL]	0.24			
	ω	0.29			
	φ [1/h]	0.030			
**Model g - dd. RBC decay**	β^*[c/mLh]	2.0e-10 - 9.2e-10	EC_{50} [ng/mL]	1.1e2	80
	H_0*	0.40 – 0.65	E_{max} [1/h]	0.093	0.33
	r	22.8	x [1/h]	Cl_Y=0.068	k_R=0.013
	ω	0.25			
	χ_{max} [1/h]	0.018			
	$k\chi_{,50}$[10^{10}c/mL]	1.05			
	γ_{max} [1/h]	0.055			
	$K\gamma_{50}$[10^{10} c/mL]	0.10			
	φ [1/h]	0.027			
**Model h - human RBC**	β^*[c/mLh]	2.1e-10 - 8.8e-10	EC_{50} [ng/mL]	1.1e2	77
	H_0*	0.40 - 0.65	E_{max} [1/h]	0.082	0.30
	r	22	x [1/h]	k_R=0.073	k_R=0.013
	ω	0.36			
	γ_{max} [1/h]	0.067			
	$k\gamma_{50}$[10^{10} c/mL]	0.20			
	λ[1/h]	0.008			
	φ[1/h]	0.040			
**Model i - exponential**	P_0 [log(P)]	-1.03 - 1.52	EC_{50} [ng/mL]	32	213
	p_{gr} [1/h]	0.16 - 0.30	E_{max} [1/h]	0.12	0.67
			x [1/h]	Cl_Y=0.025	k_R=0.020

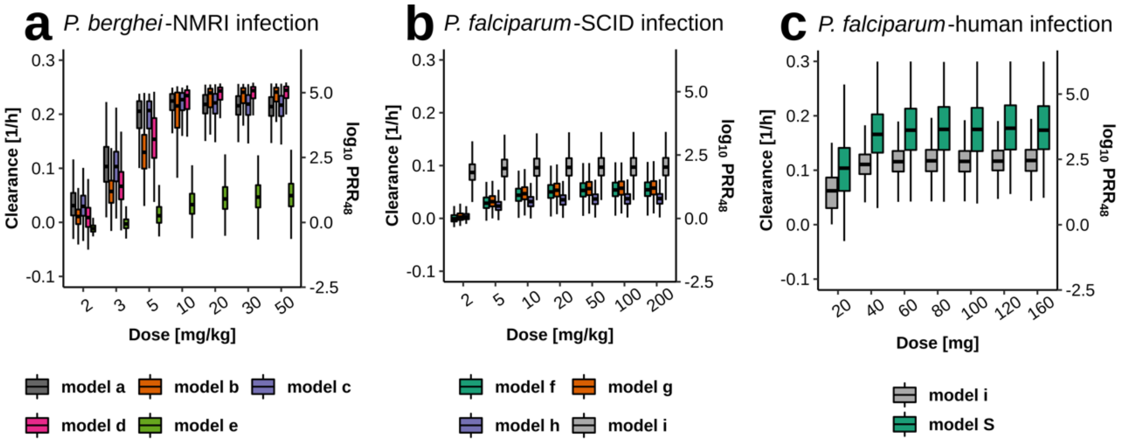

Figure S13: Parasitic clearance rates across the clinical development stages after single dose treatment with MMV048. Parasite clearance rates for *P. berghei* (a), *P. falciparum* in SCID (b) and *P. falciparum* in VIS (c) were calculated from simulation output using the methodology provided in (2). The difference in predicted clearance of *model e (reticulocytes)* for *P.berghei*-NMRI infection is caused by a difference in structural PD model (turnover) in comparison to *model a-d* (clearance). Because *model e* does not take the delayed clearance of dead parasites into account, it estimates slower parasite killing. In contrast, *model a-d* measure the actual killing of the drug and therefore higher clearance values. Delayed parasite recrudescence in lower doses influenced the estimation of parameters of drug action for *model i (exponential)* in *P. falciparum*-SCID infection, shifting them to higher drug efficacy estimates (lower EC_{50})(1). (47). For comparability a conversion to $\log_{10}$ PRR_{48} was added as secondary y-axis where $\log_{10}$ $PRR_{48} = \log_{10}(e^{\text{parasite clearance rate x 48}})$.

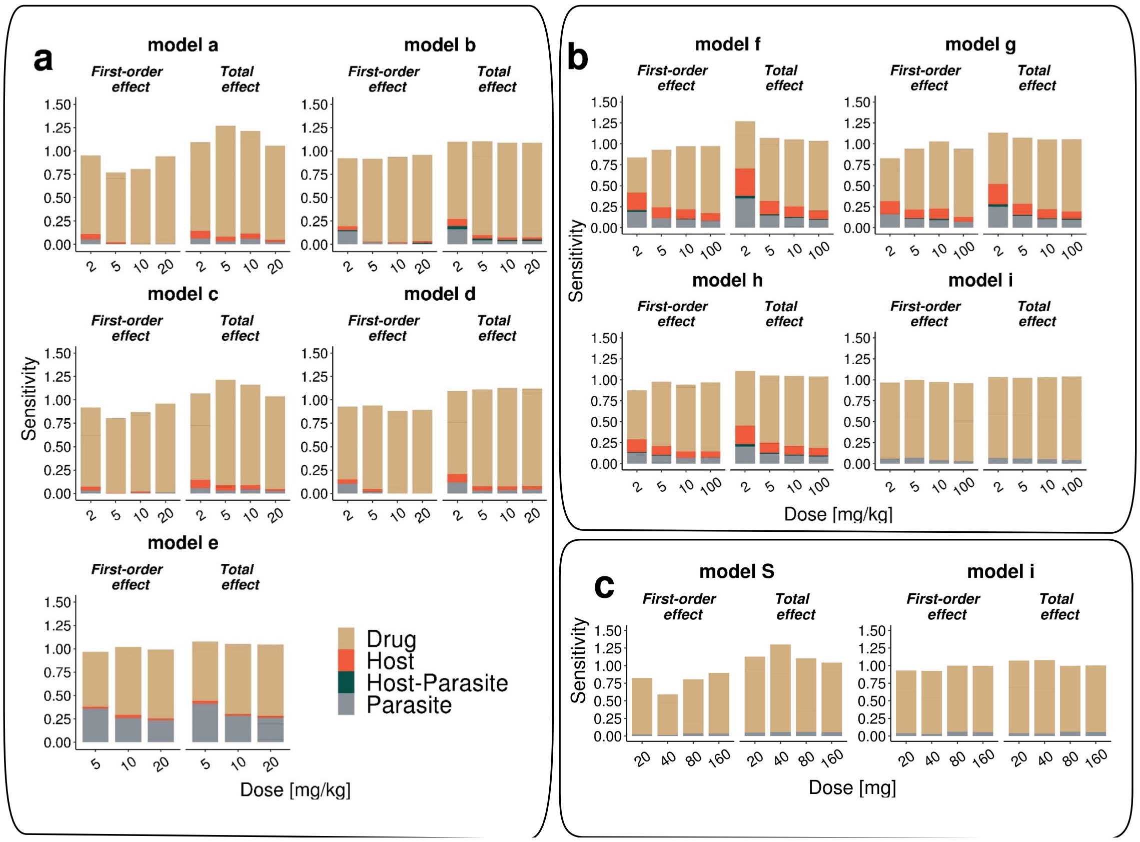

Figure S14: Sobol sensitivity analysis of parasite clearance after MMV048 treatment towards parameters of host, parasite, host-parasite, and drug dynamics for (a) *P. berghei* – NMRI , (b) *P. falciparum* – SCID, and (c) *P. falciparum*- human infection. In *P. berghei*-NMRI infection, parasite clearance after medium-high doses is highly sensitivity towards parameters of drug dynamics with the exception of *model e*. Here, the number of merozoites r and the preference for reticulocytes ε account for up to 40% of variance. Sobol indices could not be calculated for the 2 mg/kg dose group of *model e*, since the number of samples with quantifiable clearance was to small. Drug induced clearance in *P. falciparum*-SCID infections exhibits similar behavior for all mechanistic parasite growth *models f-h*. No noticeable influence of parasite and host parameters was found for *P. falciparum*-human infection. The first order effects measure individual parameter contributions and the total effect indices summarize individual and interactive parameter contributions to the outcome variance. The full set of individual parameter contributions can be found in Supplementary File 2.

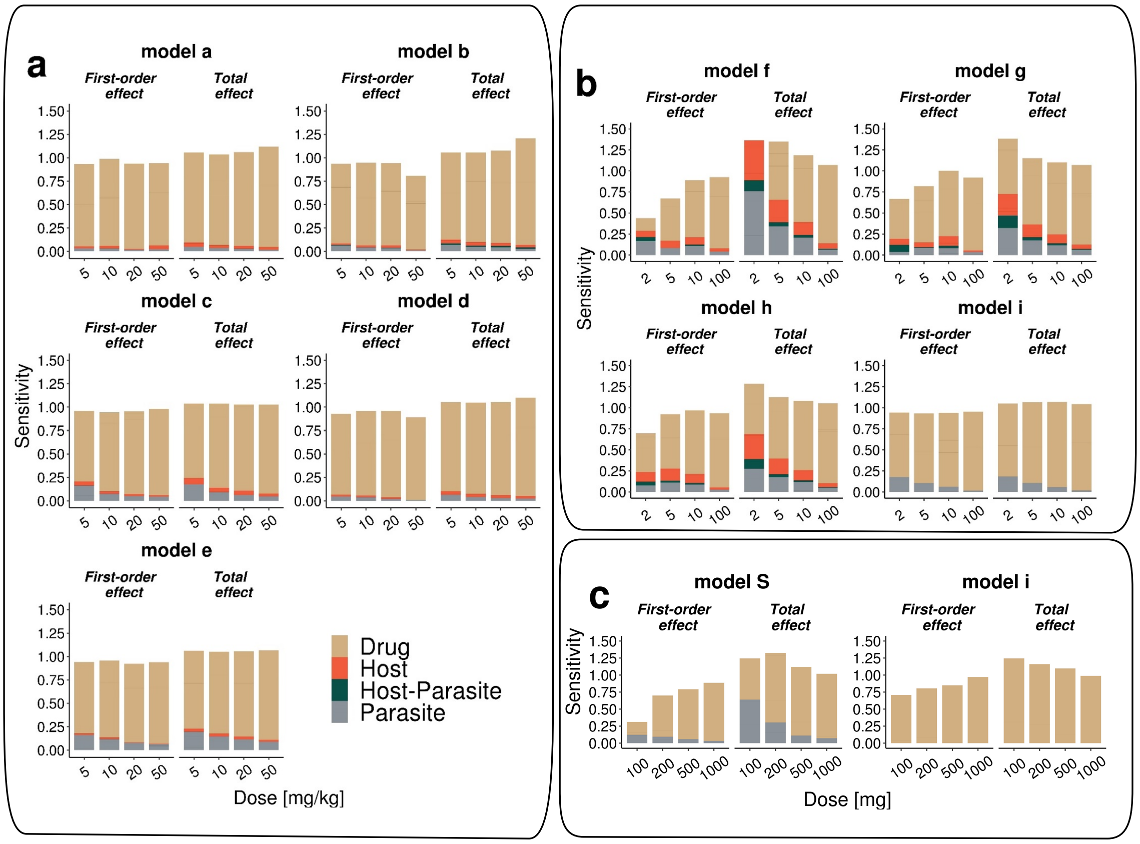

Figure S15: Sobol sensitivity analysis of parasite clearance after OZ439 treatment towards parameters of host, parasite, host-parasite, and drug dynamics for (a) *P. berghei* – NMRI , (b) *P. falciparum* – SCID, and (c) *P. falciparum*- human infection. In *P. berghei*-NMRI infection, parasite clearance after medium-high doses is highly sensitivity towards parameters of drug dynamics. Drug induced clearance in *P. falciparum*-SCID infections exhibits similar behavior for all mechanistic parasite growth *models f-h*, with lower dose ranges showing an increased sensitivity towards host and parasite parameters. *Model S* of *P. falciparum*-human infection shows an increased total effect of parasite parameters (see Discussion). No noticeable influence of parasite and host parameters was found for the exponential growth *model i*. The first order effects measure individual parameter contributions and the total effect indices summarize individual and interactive parameter contributions to the outcome variance. The full set of individual parameter contributions can be found in Supplementary File 2.

1. Burgert L, Rottmann M, Wittlin S, Gobeau N, Krause A, Dingemanse J, Möhrle JJ, Penny MA. 2020. Ensemble modeling highlights importance of understanding parasite-host behavior in preclinical antimalarial drug development. Sci Rep 10:4410.

2. Flegg JA, Guerin PJ, White NJ, Stepniewska K. 2011. Standardizing the measurement of parasite clearance in falciparum malaria: the parasite clearance estimator. Malar J 10.
